# *N,N*‐Dimethylhydrazine as a Reversible Derivatization Agent to Promote the Hydroxymethylation of Furfural with Formaldehyde

**DOI:** 10.1002/cssc.202500318

**Published:** 2025-04-04

**Authors:** Sarah Behloul, Zhen Yan, Karine De Oliveira Vigier, Frederic Guégan, François Jérôme

**Affiliations:** ^1^ Institut de Chimie des Milieux et Matériaux de Poitiers Université de Poitiers CNRS 1 rue Marcel Doré 86073 Poitiers France; ^2^ Eco‐Efficient Products and Process Laboratory Syensqo/CNRS 3966 Jin Du Rd., Xin Zhuang Industrial Zone Shanghai 201108 China

**Keywords:** acid catalysts, biomass, furfural, 5‐hydroxymethylfurfural, hydroxymethylation

## Abstract

In this report, the synthesis of 5‐hydroxymethylfurfural from concentrated feeds of two low‐cost and industrially abundant chemicals: Furfural and formaldehyde is explored. By adjusting the acidity of the solvent, an alternative mechanism is discovered in which the reaction selectivity stops to the hydroxymethylation step, in contrast to previously reported acid‐catalyzed pathways leading to the formation of the bisfuranic dimer as a major product. One of the keys of this study relies on the reversible derivation of the –CHO group of furfural with *N,N*‐Dimethylhydrazine which plays a dual role: (1) it restores the nucleophilicity of the furan ring and (2) it reacts with HCHO to form in situ an electrophilic zwiterrionic species stabilized through hydrogen transfer. By means of experimental and theoretical investigations, this reaction is optimized and it is discovered that guaiacol can be used as a bio‐based and safe solvent. Under optimized conditions, the hydroxymethylation of the furan ring of furfural occurs with more than 95% selectivity, at only 50 °C and with a stoichiometric amount of HCHO. A concentrated feed of furfural as high as 40 wt% in guaiacol can be employed without impacting the reaction selectivity, leading to an improvement of the reactor productivity to about 25 kg m^−3^ h^−1^. The recovery of the reaction products and the recycling of the *N,N*‐dimehylhydrazone are also discussed.

## Graphical abstract

The reversible derivation of the –CHO group of furfural with *N,N*‐Dimethylhydrazine not only restores the nucleophilicity of the furan ring toward formaldehyde but also opens an alternative mechanism to the classical acid‐catalyzed pathway for the selective hydroxymethylation of the furanic ring of furfural.

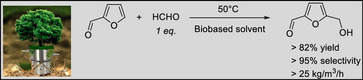

## 1 Introduction

To tentatively reduce its CO_2_ emissions, the chemical industry is now progressively replacing, to some extent, fossil‐based building blocks by bio‐based alternatives. Among these, 5‐hydroxymethylfurfural (HMF) has attracted considerable attention over the past 20 years. Indeed, HMF is considered as a promising chemical platform for the production of industrially relevant downstream chemicals such as diformyl furan, 2,5‐furandicarboxylic acid, 2,5‐bis(aminomethyl)furan, tetrahydrofuran derivatives, carboxylic acids, lactones, among many others.^[^ ^1^, ^2^, ^3^, ^4^ ^]^ HMF is typically obtained by the acid‐catalyzed dehydration of naturally abundant C6 sugars, such as glucose or fructose.^[^ ^5^ ^]^ Despite extensive research on the synthesis of HMF from sugars, the industrial implementation of these routes still faces challenges due to a lack of selectivity control, primarily due to side reactions between HMF and sugars, as well as the uncontrolled in situ self‐condensation of HMF under acid conditions, resulting in the formation of tar‐like substances, also called humins.^[^ ^4^, ^5^ ^]^ The selectivity to HMF can be increased by diluting carbohydrates in dimethylsulfoxide or in water, typically below 5 wt%.^[^ ^5^, ^6^ ^]^ When water is used, HMF is often continuously extracted with an organic solvent to further improve the selectivity. While these methods can achieve moderate to good HMF yields (>70%), these strategies unfortunately lead to expensive downstream purification processes and low reactor productivities, which are unfortunately not compatible with the industrial specifications of the field. As a result, despite its promising potential, HMF is still considered as the “sleeping giant” of the bio‐based chemistry.

In contrast to HMF, furfural, derived from C5 sugars, is commercially available on a large scale (300 kT year^−1^) and at an attractive market price (1.5–2.5 $ kg^−1^).^[^ ^7^ ^]^ However, unlike HMF, furfural has the disadvantage of being monofunctionalized, which considerably limits its application, particularly in the synthesis of polymer, the largest market share of the chemical industry.^[^ ^8^ ^]^ So far, industrial reactions have primarily targeted the hydrogenation/hydrogenolysis of C=C and C=O bonds, leading to the production of methyltetrahydrofuran and furfuryl alcohol, respectively.^[^ ^9^, ^10^ ^]^ To address this limitation, the synthesis of HMF through the hydroxymethylation of furfural with formaldehyde has been proposed. From a practical point of view, it appears as a promising pathway because (1) it couples two cheap raw materials in a 100% atom‐economical fashion and (2) it avoids possible side reactions of HMF with sugars, one of the pathways to humins formation. However, turning now to the scientific point of view, this reaction is also very challenging. Indeed, as compared to simple furan or furfural‐derived chemicals such as furfuryl alcohol or furfuryl ether,^[^ ^11^ ^]^ the presence of an electron‐withdrawing –CHO group at the C‐2 position drastically reduces the reactivity (nucleophilicity) of the furan ring of furfural toward electrophiles, such as HCHO in our case.^[^ ^12^ ^]^

**Scheme 1:**
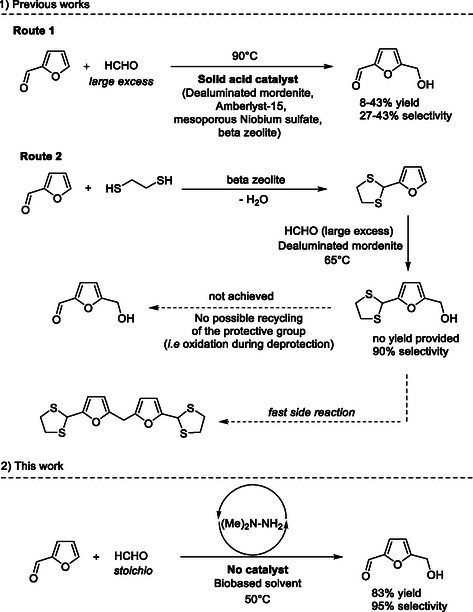
Contribution of the present work to the state of the art.

Moreau and co‐workers reported a pioneer work on the hydroxymethylation of furfural with HCHO using dealuminated mordenite as a catalyst (**Scheme** **1**, route 1).^[^ ^13^ ^]^ To facilitate the reaction of furfural with HCHO, furfural was diluted in a very large amount of HCHO (0.5 wt% furfural in formalin). However, even under these dilute conditions, the yield to HMF was only 8% at ≈30% conversion. Later, Ebinati et al. revisited this reaction using Amberlyst‐15 as a strongly acid solid catalyst, resulting in a 43% yield in HMF at 75% conversion.^[^ ^14^ ^]^ However, once again, these improvements were only obtained under highly dilute conditions. Similar conclusions were drawn from the work of Shibata et al.^[^ ^15^ ^]^ Cao et al.^[^ ^16^ ^]^ and more recently Liu et al.^[^ ^11a^ ^]^ who used zeolites and mesoporous niobium phosphate in both batch and continuous modes.

Recently, through combined experimental and theoretical investigations, we and others have shown that the nucleophilicity of the furan ring of furfural could be restored by derivatization of the –CHO group with acetals, 1,2‐ethanedithiol, *N,N*‐dimethylhydrazine, among others.^[^ ^17^, ^18^, ^19^, ^20^, ^21^ ^]^ This strategy has successfully been reported in Diels–Alder reactions with furfural, a reaction which is thermodynamically unfavorable when the –CHO group is not derivatized.^[^ ^18^, ^20^, ^21^ ^]^ To the best of our knowledge, Moreau et al. reported the first, and unique report, on the catalytic hydroxymethylation of furfural derivatized with 1,2‐ethanedithiol, using dealuminated mordenite as solid acid catalysts (Scheme 1, route 2).^[^ ^13^ ^]^ As expected, the protection of the –CHO group has increased the selectivity of the hydroxymethylation reaction to 90%, albeit here again with a 60 vol% excess of formalin. The use of an excess of formalin was actually required to prevent the fast double condensation of the in situ as‐formed intermediate protected HMF with another molecule of furfuryl dithiane, leading to the formation of a dimer. Indeed, the intermediate protected HMF is much more reactive than HCHO and the double condensation is much faster, making difficult the control of the reaction selectivity to HMF.

As stated by the authors, the thioacetal group may be removed at the end of the reaction, even though it was not attempted in their research. Recycling this protective agent is highly challenging due to the oxidative conditions required for deprotection, which lead to the oxidation of sulfur atoms.^[^ ^22^, ^23^, ^24^, ^25^ ^]^ Given the cost of 1,2‐ethanedithiol, this limitation prohibitively increases the production cost of this pathway, as we have previously demonstrated.^[^ ^19^ ^]^

Being able to (1) activate furfural through a reversible derivatization and (2) selectively stop the reaction to the hydroxymethylation step (i.e., HMF) is a challenging scientific task which we address in this study. By tackling this scientific challenge, we discovered and exploited in this work a synergistic effect between *N,N*‐dimethylhydrazine, which acts as a reversible protective agent for the concomitant activation of the =CH bond of furfural and HCHO, and guaiacol as a bio‐based solvent to selectively stop the reaction to the hydroxymethylation step. In contrast to previous routes, this work has the advantage to couple concentrated feed of furfural (>25 wt%) with a stoichiometric amount of HCHO and at only 50 °C, leading to the formation of HMF with more than 95% yield.

## 2 Results and Discussion

### 2.1 Derivatization of Furfural and Hydroxymethylation

As a reference reaction, furfural was first reacted with a stoichiometric amount of HCHO (formalin, 37 wt% HCHO in water) at 50 °C. As expected, with (0.1 eq. H_2_SO_4_) or without acid catalyst, no hydroxymethylation reaction occurred, confirming the low reactivity of furfural toward electrophiles. Instead, furfural was decomposed to a mixture of different unidentified chemicals, mostly tar‐like materials, especially when H_2_SO_4_ was used as a catalyst. Following our previous strategy, furfural was then protected with *N,N*‐dimethylhydrazine to increase the electronic density, and thus the nucleophilicity, of the furan ring of furfural.^[^ ^18^, ^19^ ^]^ The choice for *N,N*‐dimethylhydrazine as a protective group was motivated by (1) a reversible and convenient deprotection of furfural at the end of the reaction, allowing the recycling of *N,N*‐dimethylhydrazine (discussed later) and (2) the higher stability of furfural‐hydrazone than furfural‐dioxolane under acid conditions.^[^ ^19^, ^26^ ^]^ In addition, previous works on Diels‐Alder^[^ ^18^, ^20^, ^27^ ^]^ and Michael additions^[^ ^28^, ^29^ ^]^ revealed that the derivatization of the –CHO group of furfural with *N,N*‐dimethylhydrazine significantly increased the highest occupied molecular orbital coefficient at the C5 position of the as‐formed furfural‐hydrazone. This result can be understood by the delocalization of the nitrogen atom's lone pair into the π‐system of the furan ring. Taking advantage of this electronic effect occurred to us as a possible mean to improve the reactivity of the =CH bond of furfural toward an electrophile such as HCHO.

In a typical experiment, furfural was reacted with 1.2 eq. of *N,N*‐dimethylhydrazine in ethanol, without any catalyst, affording the corresponding furfuryl‐hydrazone (**2**) in a quantitative yield, as previously described by Kamimura and co‐workers (ESI, Figure S6, Supporting Information).^[^ ^30^ ^]^ After the removal of ethanol, the as‐obtained furfural‐hydrazone (**2**) was then reacted without any further purification with a stoichiometric amount of HCHO (formalin, 37 wt% HCHO in water) at 50 °C (**Table** **1**). In the absence of any acid catalyst, 60% of furfuryl‐hydrazone (**2**) was converted after 24 h of reaction, affording the desired HMF‐hydrazone (**3**) in a 26% yield (i.e., 43% selectivity) (Table 1, entry 1). Full characterizations of the HMF‐hydrazone (**3**) are provided in Figure S7, Supporting Information. Although the reaction conditions were not optimized, this result highlights the capacity of the hydrazone moiety to activate the furan ring of furfural. Unfortunately, under these conditions, the selectivity of this reaction was still not satisfactory. In particular, due to the reasons discussed above, the bisfuranic adduct (**4**), resulting from the double condensation of furfuryl‐hydrazone (**2**) on HCHO, was formed in 10% yield. Full characterizations of the dimer (**4**) are provided in Figure S8, Supporting Information. Deprotected furfural was also observed in about 10% yield in this experiment. Other co‐products (24%) mainly consist of tar‐like products, as also corroborated by the deep darkening of the reaction media over time. Efforts to limit the formation of the bisfuranic adduct (**4**) by diluting the reaction mixture with water (10 wt% of furfuryl‐hydrazone (**2**) in water) decreased the rate of the reaction by a factor of 1.7 but, however, did not improve the selectivity (Table 1, entry 2).

**Table 1:** Impact of solvent in the hydroxymethylation of furfural‐hydrazone derivative 2 with formaldehyde.a)

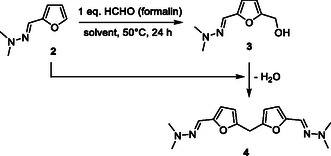				
Entry	Solvent	Conv. 2 (%)	Yield 3 (%)	Yield 4 (%)
1	None	60	26	10
2	Water	35	16	7
3	Glycerol	36	21	8
4	Ethylene glycol	20	10	7
5	Methanol	9	1	0
6	Ethanol	8	0	0
7	Isopropanol	8	0	0
8	Acetonitrile	5	0	0
9	Dimethylformamide	2	0	0
10	Diglyme	5	0	0
11	Water/methanol(55/45)	38	18	5
12	Water/isopropanol(55/45)	46	20	10
13	Trifluoroethanol	94	63	12
14	HFIPb)	83	82	0
15	Choline chloride/urea(1:2)	7	0	0
16	Choline chloride/glycerol(1:1.5)	8	3	1

a) Furfural‐hydrazone (mmol, 10 wt% V^−1^), aqueous formaldehyde 37% (1 eq.), solvent, 50 °C, 24 h. Yield and selectivity were estimated according to NMR quantification (ESI);

b) Yield estimated after 15 min (maximum according to kinetic study, Figure S14, Supporting Information).

In these preliminary experiments, it is noteworthy that furfural‐hydrazone (**2**) is not entirely miscible with formalin (aq. 37 wt% HCHO), which may be one of the reasons for the limited selectivity to HMF‐hydrazone (**3**). Hence, addition of different polar solvents was attempted to homogenize the reaction medium and to facilitate a better interaction between furfural‐hydrazone (**2**) and HCHO. In such experiments, the concentration of furfuryl‐hydrazone (**2**) was maintained to 10 wt% for consistency with industrial reactor productivity standards. Other reaction parameters remained unchanged (50 °C, furfuryl‐hydrazone (**2**)/ HCHO molar ratio = 1).

In polar solvents such as alcohols, acetonitrile, dimethylformamide, diglyme, and mixtures of water and alcohols, the conversion of furfuryl‐hydrazone (**2**) and the yield of the desired HMF‐hydrazone (**3**) remained below 36% and 21%, respectively, after 24 h of reaction (Table 1, entries 3–13) (Figure S9–S14, Supporting Information). Among these polar solvents, it should be noted that polyols such as glycerol and ethylene glycol provided the best results in terms of conversions and yields (Table 1, entries 3, 4). More information on this aspect is provided later. In stark contrast, perfluorinated solvents, such as 1,1,1,3,3,3‐hexafluoropropanol (HFIP) and trifluoroethanol, markedly improved both the conversion and the selectivity (Table 1, entries 13–14). In particular, in HFIP, 83% of the furfuryl‐hydrazone (**2**) were converted after only 15 min and the reaction was nearly fully selective to the desired HMF‐hydrazone (**3**) (selectivity= 99%). Note that extending the reaction time to 30 min led to 90% conversion of furfuryl‐hydrazone (**2**) without significantly impacting the selectivity to HMF‐hydrazone (**3**) (>96%).

Despite these very promising results, the utilization of HFIP as a reaction solvent unfortunately raises major environmental concerns. As a result, the replacement of HFIP by a safer solvent is mandatory. To guide us in this direction, we conducted a mechanism investigation to tentatively elucidate the origin of this remarkable selectivity observed in the presence of HFIP.

### 2.2 Mechanism Investigation

**Figure 1:**
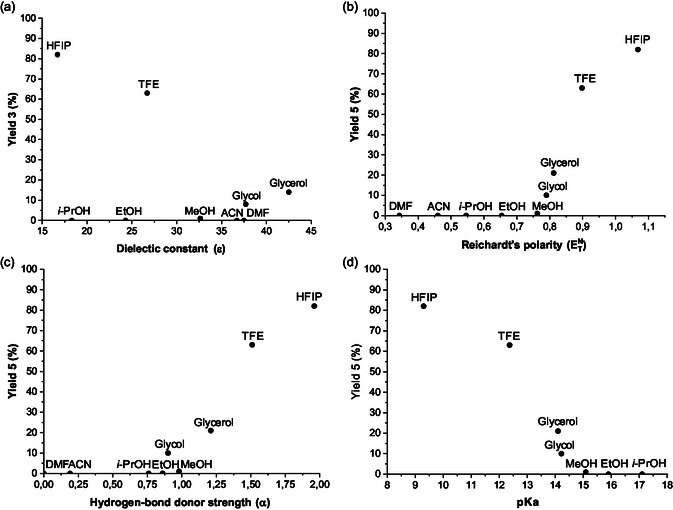
Yield correlation to a) dielectric constant, b) Reichardt's polarity, c) HBD strength, and d) Brønsted acidity. Yields were all collected after 24 h of reaction, except for TFE and HFIP (15 min).

First, results collected in Table 1 were tentatively correlated with the physicochemical properties of solvents used. To this end, the yield to the HMF‐hydrazone (**3**) was plotted as a function of the solvents dielectric constant (ε),^[^ ^31^, ^32^ ^]^ Reichardt's polarity ($E_{T}^{N}$),^[^ ^33^ ^]^ hydrogen‐bond donor (HBD) strength (according to Kamlet–Taft scale (α)),^[^ ^33^ ^]^ and Brønsted acidity^[^ ^33^ ^]^ using data from the literature (**Figure** **1**).

No satisfying correlation was observed between the reaction yield/selectivity and the dielectric constant. However, a correlation was observed considering hydrogen bond strength, Reichardt's polarity ($E_{T}^{N}$), and Brønsted acidity as parameters.

To discern the dominant factor between polarity (hydrogen bond strength, Reichardt's polarity ($E_{T}^{N}$) as descriptors) and acidity, another set of experiments was conducted in polar solvents characterized by strong hydrogen bond networks but low acidity. To this end, the hydroxymethylation of furfuryl‐hydrazone (**2**) was performed in two different deep eutectic solvents (DESs) such as choline chloride/ glycerol and choline chloride/ urea (Table 1, entries 15‐16). DESs are typically composed of a HBD and a hydrogen bond acceptor, forming an extensive hydrogen bond network.^[^ ^34^, ^35^ ^]^ Interestingly, in both DESs, furfuryl‐hydrazone (**2**) was converted in a very low amount (< 8%, after 24 h of reaction), suggesting that hydrogen bond strength cannot explain alone the remarkable results collected in HFIP. Instead, these results suggest that Brønsted acidity is presumably one of the plausible explanations behind the greatest selectivity observed in HFIP.

To explore more deeply the impact of the acid properties of solvents on the selectivity to HMF‐hydrazone (**3**), solvents listed in Table 1 were retested in the presence of a catalytic amount of H_2_SO_4_ (10 mol%). Results are summarized in **Table** **2**. All data were provided at 50% conversion to accurately compare all entries together. Additional data (i.e., yields and conversion over time for each solvent) are provided in the ESI (Figure S15–S24, Supporting Information).

**Table 2:** Hydroxymethylation of furfural‐hydrazone derivative 2 on formaldehyde using H_2_SO_4_ in various solvents.

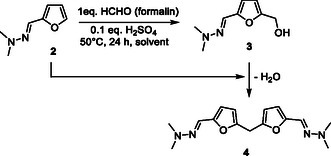				
Entry	Solvent	Yield 3 (%)	Select. 3 (%)	Yield 4 (%)
1	Nonea)	4	8	20
2	Glycerol	0	0	17
3	Ethylene glycol	0	0	28
4	Methanol	0	0	38
5	Ethanol	0	0	34
6	Isopropanol	0	0	30
7	Acetonitrile	0	0	36
8	Dimethylformamide	0	0	24
9	Trifluoroethanol	6	12	44
10	HFIP	41	82	9

a) Furfural‐hydrazone (mmol, 10 wt% V^−1^), aqueous formaldehyde 37% (1 eq.), H_2_SO_4_ (10% mol), solvent, 50 °C, 24 h results were collected at 50% conversion. Yield and selectivity were estimated according to NMR quantification (ESI);

Addition of a catalytic amount of H_2_SO_4_ (10 mol%) in solvent‐free conditions or in polar solvents such as alcohols, acetonitrile, and dimethylformamide mainly drove the selectivity of the reaction to the bisfuranic adduct (**4**). As shown in Table 2, in these acid conditions, the bisfuranic adduct (**4**) was formed in 17%–38% yield while the targeted HMF‐hydrazone (**3**) was no longer observed (Table 2, entries 1–8). Similar results were observed using other acid catalysts including Brønsted acids (formic acid, hydrochloric acid, acetic acid, trifluoroacetic acid) and Lewis acids (aluminum chloride, bismuth triflate) in ethanol (Table S3, Supporting Information). In all cases, a monitoring of the reaction over time by ^1^H NMR revealed the formation of the bisfuranic adduct (**4**) from the initial stage of the reaction. This is in perfect agreement with previous reports, suggesting that, under acidic‐catalyzed conditions, the bisfuranic adduct (**4**) is formed as a dominant product.^[^ ^13^ ^]^ In fluorinated solvents, results were different. In trifluoroethanol, the targeted HMF‐hydrazone (**3**) was observed in 6% yield (i.e., 12% selectivity), albeit once again the bisfuranic adduct (**4**) was the major product formed (10%–44% selectivity) (Table 2, entries 9).

However, more surprisingly, when 0.1 eq. of H_2_SO_4_ was added to HFIP, the desired HMF‐hydrazone (**3**) product still remained formed as a dominant product (82% selectivity vs 9% for the bisfuranic adduct (**4**) (Table 2, entry 10). All these results being compared at isoconversion (50%), this observation strongly suggests that the reaction mechanism taking place in HFIP differs from the classical acid‐catalyzed one occurring in other organic solvents, otherwise addition of H_2_SO_4_ in HFIP would have shifted the reaction selectivity toward the bisfuranic derivatives (**4**).

To go one step further in understanding the reaction mechanism taking place in HFIP, the reaction was monitored by ^1^H NMR. Beside the characteristic peaks corresponding to the starting furfural‐hydrazone (**2**) and the as‐formed HMF‐hydrazone (**3**), four additional peaks were clearly observed in the baseline of the NMR spectrum (Figure S25, Supporting Information). Three of them, and with a similar integration, were located in the =CH region and another one was in the region of the –CH_2_OH. A monitoring of the reaction over time by ^1^H NMR showed that these four peaks appear at the beginning of the reaction and then disappeared as long as the HMF‐hydrazone (**3**) is formed, suggesting the formation of intermediate species in HFIP. This intermediate formed in HFIP has a ^1^H NMR spectrum signature very similar to that of the HMF‐hydrazone (**3**), except that all peaks are shifted by about 0.07 ppm. In a first approach, this could be explained by the *Z*/*E* isomerization of the C=N bond, but this hypothesis was rapidly ruled out by the work of Gómez‐Zavaglia et al. showing that the hydrazone moiety existed exclusively in a *E* configuration (the *Z* configuration is about 30 kcal mol^−1^ higher in energy).^[^ ^36^ ^]^ It is worth noting that this intermediate was not observed by ^1^H NMR when the reaction was performed in alkyl alcohols instead of HFIP, further supporting that an alternative reaction pathway occurs in HFIP.

The furfuryl‐hydrazone (**2**) is not only a base but also a good nucleophile.^[^ ^37^, ^38^ ^]^ Hence, we hypothesized that this intermediate product observed in HFIP could result from the nucleophilic addition of the hydrazone moiety to HCHO to form a zwitterionic species, which is an isomer of HMF‐hydrazone (**3**). This hypothetical reaction pathway was further supported by reacting the furfural‐hydrazone (**2**) with n‐butyl bromide, leading to the formation of the hydrazonium salt which was detected by ^1^H NMR and HRMS (Figure S26, Supporting Information, ESI). This result confirms the nucleophilic nature of the hydrazone moiety, in line with previous report.^[^ ^38^ ^]^ The impact of solvent can then be further rationalized. As shown above, acidity seems to be a significant factor to explain the peculiarity of HFIP in this reaction. One may surmise that the zwitterionic intermediate could rather be destabilized, unless placed in a polar and protic environment (stabilization of the alkoxide moiety by a strong H bond or even a proton transfer). DFT calculations confirmed that the result of the addition of HCHO on the hydrazone moiety could indeed be stabilized by a proton transfer from HFIP, while the species spontaneously decomposes back into (**2**) and HCHO when interacting with a weaker acid such as H_2_O or methanol, or in the absence of any solvent molecule. Computational details and optimized geometries are provided in the ESI.

**Scheme 2:**
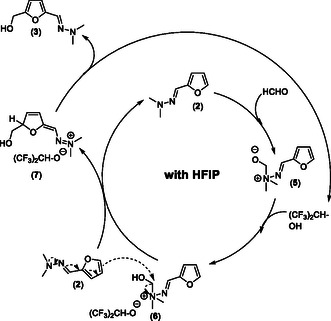
Proposed reaction mechanism pathway in the presence of HFIP.

On the basis of all these results, a plausible reaction mechanism can be proposed to rationalize the unexpected selectivity to HMF‐hydrazone (**3**) observed in HFIP (**Scheme** **2**). In such fluorinated solvent, the furfuryl‐hydrazone (**2**) first reacts with HCHO, through nucleophilic addition, to form the zwitterionic species (**5**). This species is then protonated by HFIP to form the intermediate (**6**). This latter then reacts with another molecule of furfuryl‐hydrazone (**2**) to form the intermediate species (**7**) and regenerate (**2**). Intermediate (**7**) then releases one proton to form the HMF‐hydrazone (**3**) and regenerate HFIP. Satisfactorily, further DFT calculations showed the transfer of HCHO from intermediate (**6**) and an equivalent of (**2**) should proceed quite easily, with an activation barrier of 23.9 kcal mol^−1^ at ambient conditions (comparable to the activation barriers we previously reported for the endocyclic additions of formaldehyde to thioacetal‐protected furfural).^[^ ^19^ ^]^

**Figure 2:**
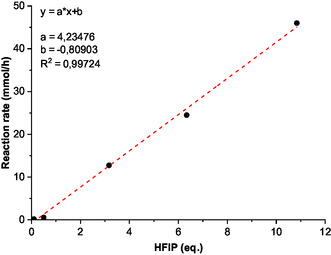
Impact of the amount of HFIP on the reaction rate.

In such mechanism, HFIP is expected to act as a catalyst. Hence, to further support the proposed reaction pathway, the amount of HFIP was reduced to assess the impact on the reaction rate and selectivity to HMF‐hydrazone (**3**). Results are shown in **Figure** **2**. In agreement with the proposed mechanism, when the amount of HFIP was gradually decreased from 11 eq. to only 0.05 eq., a decrease of the reaction rate was observed, and that without impacting the selectivity to HMF‐hydrazone (**3**), at least over 3 eq. of HFIP. Indeed, below 3 eq. of HFIP, the H2O (i.e., formalin)/HFIP molar ratio becomes too high, which impacted the solubility of the furfuryl‐hydrazone (**2**) and thus the selectivity of the reaction. These results strongly support the catalytic role of HFIP (Figure S27–S31, Supporting Information).

**Scheme 3:**
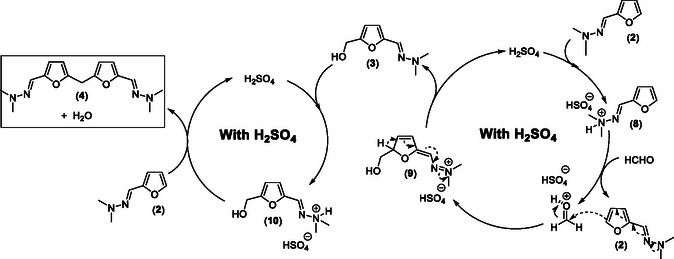
Proposed reaction mechanism pathway in the presence of H_2_SO_4_.

In such proposed reaction mechanism, one of the keys is the formation of the zwitterionic species (**5**). In HFIP, this intermediate is viable only because the conjugated base of HFIP (i.e., (CF3)2CH–O‐) is less basic than the zwitterionic species (**5**), as hinted by the spontaneous deprotonation of HFIP observed in the DFT calculations. In the case of H2O or MeOH, the corresponding conjugated bases HO‐ and MeO‐, respectively, are more basic than the zwitterionic species (**5**) making impossible the formation of this latter. Hence, in conventional solvents, a classical acid‐catalyzed mechanism takes place. In such solvents, this reaction is rather slow but it can be accelerated by addition of a catalytic amount of H_2_SO_4_. In this scenario, the furfuryl‐hydrazone (**2**) is first protonated to form the furfuryl‐hydrazonium salt (**8**) which acts as an acid catalyst to activate HCHO, which promotes the nucleophilic addition of a second molecule of furfuryl‐hydrazone (**2**) to form the intermediate (**9**) (**Scheme** **3**). This latter then affords the desired HMF‐hydrazone (**3**) by regenerating H_2_SO_4_. Under acid conditions, and as previously reported by others and confirmed by us in this study, the –CH2OH group of HMF‐hydrazone (**3**) is much more reactive than HCHO, due to the stabilization of a formal carbocation in α‐position of the furan ring, leading to a very rapid nucleophilic addition of a second molecule of furfuryl‐hydrazone (**2**) to the in situ protonated HMF‐hydrazone (**10**) yielding the bisfuranic adduct (**4**) as a major product and regenerating H_2_SO_4_ (Scheme 3). This acid‐catalyzed reaction pathway also rationalizes the much higher conversion rates observed in Table 1 (entries 3, 4) when using polyols such as glycerol and ethylene glycol. Indeed, the p*K*_a_ values of glycerol and ethylene glycol are slightly lower than those of methanol, ethanol, or isopropanol which may explain the reaction smoothly proceeded in these two polyols without addition of a catalytic amount of H_2_SO_4_.

Finally, to further support these two different reaction pathways, the conversion rates of furfuryl‐hydrazone (**2**) and HMF‐hydrazone (**4**) were independently determined in HFIP and in ethanol containing a catalytic amount of H2SO_4_ (**Table** **3**). In HFIP, furfuryl‐hydrazone (**2**) was converted to HMF‐hydrazone (**3**) at a 45 mmol/h rate, which was found more than 200 times higher than the conversion rate of HMF‐hydrazone (**3**) to the bisfuranic adduct (**4**), thus rationalizing the remarkable selectivity to HMF‐hydrazone (**3**) observed in HFIP (Table 3, entries 1, 2). Conversely, in ethanol containing a catalytic amount of H_2_SO_4_, the conversion rate of HMF‐hydrazone (**3**) to the bisfuranic adduct (**4**) (10.7 mmol h^−1^) was about 13 times faster than the conversion rate of furfuryl‐hydrazone (**2**) to HMF‐hydrazone (**3**) (0.8 mmol h^−1^), explaining in such pathway the major formation of the bisfuranic adduct (**4**) (Table 3, entries 3, 4). If one may now compare the conversion rate of furfuryl‐hydrazone (**2**) in HFIP and in ethanol containing 0.1 eq. of H_2_SO_4_, it can be calculated that the reaction is about 55 times faster in HFIP than in acidified ethanol, which also explain (**1**) the fast conversion of furfuryl‐hydrazone (**2**) in HFIP (Table 1, entry 14) and (**2**) the very little impact of the addition of H_2_SO_4_ in HFIP in terms of selectivity to the desired HMF‐hydrazone (**3**) (Table 2, entry 10).

**Table 3:** Conversion rate of furfuryl‐hydrazone (2) and HMF‐hydrazone (3) in HFIP and ethanol containing 0.1 eq. of H_2_SO_4_.a)

Entry	Reaction	Solvent	Conversion rate [mmol h^−1^]
1	(2) + HCHO	HFIP	45
2	(2) + (3)	HFIP	0.22
3	(2) + HCHO	Acidified EtOH	0.83
4	(2) + (3)	Acidified EtOH	10.7

a) Reactions performed at 50 °C from a substrate concentration of 10 wt%. Data were collected by ^1^H NMR.

### 2.3 Replacement of HFIP by Bio‐Based Solvents

Based on all these results, it is now possible to identify some avenues of thought to consider the substitution of HFIP by a safer solvent. To succeed in this task, few criteria are essential to take into account. First, the solvent should exhibit an acid character to promote the reaction. However, a compromise needs to be found in terms of acid strength. Indeed, the acid strength of the solvent should not be too high to prevent the occurrence of the unselective acid‐catalyzed pathway, leading to the dominant formation of the bisfuran adduct (**4**). Second, the conjugate base of the acidic solvent should be sufficiently stabilized to promote the protonation of the zwitterionic species (**5**). Considering these criteria, it occurred to us that phenol derivatives could be potential substitutes to HFIP. Indeed, phenol derivatives exhibit Brønsted acidity comparable to that of HFIP, with pKa values ranging from 9 to 10 owing to the stabilization of the conjugate phenolate base by delocalization, which should favor the protonation of the zwitterionic species (**5**). In this context, guaiacol, 2‐ethoxyphenol, and creosol were tested as a potential substitute of HFIP. Results are summarized in **Table** **4**. Guaiacol, creosol, and 2‐ethoxyphenol led to about 80% conversion of furfuryl‐hydrazone (**2**) after 24 h of reaction. To our great delight, as anticipated from our proposed mechanism in HFIP (Scheme 2), in all cases, the reaction was highly selective to the desired HMF‐hydrazone (**3**) (selectivity >93%). Among these phenol derivatives, guaiacol afforded the best results with a yield to HMF‐hydrazone (**3**) of 83% at 87% conversion of furfuryl‐hydrazone (**2**), i.e., selectivity of 95%. In terms of selectivity, these results are comparable than those obtained in HFIP, although the time required to obtain complete conversion of furfuryl‐hydrazone (**2**) is higher in guaiacol (≈24 h) than in HFIP (<1 h). As compared to other phenol derivatives, guaiacol is of particular interest because of its renewable origin (from lignin)^[^ ^39^, ^40^ ^]^ and its low toxicity,^[^ ^41^, ^42^ ^]^ offering an environmentally friendly alternative to HFIP. It should be noted that phenol derivatives have low hydrogen bonding donor capacity (≈0.5–0.7 on the Kamlet–Taft scale),^[^ ^34^ ^]^ confirming, once again, our above‐discussed mechanism that this property of the solvent does not impact significantly the selectivity of the reaction.

**Table 4:** Hydroxymethylation of furfural‐hydrazone derivative 2 on formaldehyde in various solvents—part 2.

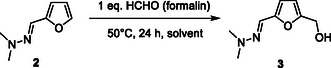			
Entry	Solvent	Conv. (%)	Yield 3 (%)
1	HFIP	83a)	82
2	3‐Methyltrifluorophenol	78	75
3	Guaiacol	87	83
4	Creosol	78	73
5	2‐Ethoxyphenol	75	67

a) Yield estimated after 1.5 min (maximum according to kinetic study).

**Figure 3:**
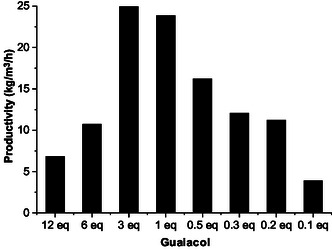
Reactor productivity as a function of the amount of guaiacol.

Further investigations were performed to optimize the reaction in guaiacol, in particular to maximize the reactor productivity which is among one of the important factors for implementation on a larger scale. **Figure** **3** illustrates the reactor productivity as a function of guaiacol equivalents. As observed above in the case of HFIP, the amount of guaiacol can be reduced from 12 to 3 eq. without impacting the selectivity to HMF‐hydrazone (**3**), resulting in an increase of the reactor productivity from 7 to 25 kg m^−3^ h^−1^. The amount of guaiacol can be further decreased to only 0.1 eq. still without any impact on the selectivity to HMF‐hydrazone (**3**), showing once again that the solvent behaves as a catalyst. However, at this low amount of guaiacol, the required time to reach complete conversion of furfuryl‐hydrazone (**2**) significantly increased, which unfortunately lowered the overall productivity of the reactor (Figure S32–S39, Supporting Information). Altogether, these results indicate that 3 eq. of guaiacol, relative to furfuryl‐hydrazone (**2**) (i.e., 40 wt% of furfuryl‐hydrazone (**2**) in guaiacol), is a good compromise to reach the highest reactor productivity, without impacting the selectivity to HMF‐hydrazone (**3**). For the sake of comparison, one should note that these productivities are about 20 times higher than those recalculated by us from previous existing reports (estimated between 0.32 and 1.22 kg m^−3^ h^−1^).^[^ ^11^, ^13^ ^–^ ^16^ ^]^

As observed in HFIP, further inspections of the reaction by ^1^H NMR also clearly highlighted the intermediate formation of the zwiterrionic species (**5**) in guaiacol (Figure S40, Supporting Information), which further support the proposed reaction mechanism in Scheme 2.

At the end of the reaction, guaiacol was conveniently separated from HMF‐hydrazone (**3**) by distillation under vacuum (35 °C, <10 − 2 mbar). Recovered guaiacol has a purity as high as 97% and could be recycled for other experiments, while HMF‐hydrazone (**3**) was recovered as a yellow‐brownish oil with a purity of 89% (contamination with remaining guaiacol). Note that liquid–liquid phase extraction of guaiacol using cyclohexane is also an efficient mean to separate (and recycled) guaiacol from the HMF‐hydrazone (**3**).

### 2.4 Downstream Process for Recovering HMF

**Scheme 4:**
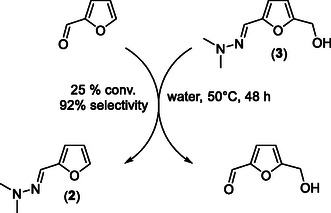
“Transhydrazonation” reaction between furfural and HMF‐hydrazone (3).

Finally, the recovered HMF‐hydrazone (**3**) could be conveniently deprotected to HMF in acidified water, as previously described in the literature.^[^ ^28^, ^29^ ^]^ The *N,N*‐dimethylhydrazine can be even potentially recycled using a biphasic acidified water/toluene solution as previously described by Bruijnincx and co‐workers^[^ ^18^ ^]^ During the course of our investigations, we discovered an alternative mean to recycle the *N,N*‐dimethylhydrazine without the need of any acid catalyst. In particular, we explored the feasibility of the catalyst‐free “transhydrazonation” reaction between HMF‐hydrazone (**3**) and furfural (**Scheme** **4**). This idea was motivated by modeling this pathway using the Spartan’18 software. Interestingly, the computed Δ*G* was predicted to be −0.8 kcal mol^−1^, suggesting that this “transhydrazonation” reaction should be feasible, at least an equilibrium is expected. To support these results, HMF‐hydrazone (**3**) was mixed with 1 eq. of furfural and heated at 50 °C in water. To our delight, after 48 h of reaction, an equilibrium was reached at which 25% of HMF‐hydrazone (**3**) and furfural were converted to 23% yield of HMF, i.e., 92% selectivity to HMF, showing the feasibility of this alternative pathway. One of the possibility to shift this thermodynamic equilibrium to HMF will consist in a continuous extraction of the as‐formed HMF or the use of an excess of furfural. This strategy is typically handled on a large scale for thermoneutral reactions. In our case, we did not explore this possibility as HMF is known to be relatively instable. Instead, after reaching the thermodynamic equilibrium (25% conversion), we opted for a separation of HMF and furfuryl‐hydrazone (**2**) by liquid–liquid phase extraction with toluene or methylisobutylketone. Albeit not optimized yet, this strategy offers the possibility to quickly extract HMF and to potentially recycle the as‐formed furfuryl‐hydrazone for another batch reaction. As compared to the classical diluted acid‐catalyzed pathway, this alternative pathway has the advantage to overcome problems often encountered on a larger scale with mineral acids such as corrosivity issues or the generation of salt waste at the end of the deprotection reaction.

## 3 Conclusion

We showed here that the (reversible) derivatization of furfural with *N,N*‐Dimethylhydrazine improves the nucleophilicity of the furan ring allowing it to react with HCHO. By adjusting the solvent acidity, we discovered an alternative mechanism to the classical acid‐catalyzed reaction, allowing us to selectively stop the reaction to the hydroxymethylation step, versus the dominant formation of a bisfuranic dimer in previous works. One of the keys relies on the nucleophilic addition of furfuryl‐hydrazone on HCHO affording a zwiterrionic species which is stabilized in weak acid solvent thanks to proton transfers. If first results were collected in HFIP, elucidation of the reaction mechanism allowed us to propose guaiacol as a bio‐based and environmentally friendlier solvent than HFIP. Under optimized conditions, the hydroxymethylation of the furan ring occurred with more than 94% selectivity, at only 50 °C and using a stoichiometric mixture of furfural‐hydrazone and HCHO. Importantly, concentrated feeds of furfuryl‐hydrazone can be used (up to 25 wt%) in guaiacol, without impacting the reaction selectivity, affording reactor productivities of about 25 kg m^−3^ h^−1^. At the end of the reaction, the as‐formed HMF‐hydrazone can be advantageously deprotected through a catalyst‐free transhydrazonation reaction upon addition of furfural, yielding deprotected HMF and regenerating an equivalent of furfuryl‐hydrazone.

From a general point of view, this work opens an access to concentrated feed of HMF from low‐cost and industrially abundant chemicals such as furfural and HCHO at 50 °C and in a nearly 100% atom economical fashion. In addition, this work contributes to provide insights, to some extent, on the magic role of HFIP often observed in acid‐catalyzed reactions.

## Supporting Information

The authors have detailed methodologies, including synthetic routes, analytical methods, NMR, and MS spectrum, kinetics data, as well as additional references, in the Supporting Information.

## Conflict of Interest

The authors declare no conflict of interest.

## Supporting information

Supplementary Material

## Data Availability

The data that support the findings of this study are available from the corresponding author upon reasonable request.
