# Supplementary material for: N,N‐Dimethylhydrazine as a Reversible Derivatization Agent to Promote the Hydroxymethylation of Furfural with Formaldehyde
Source: ChemSusChem. 2025 Apr 4;18(12):e202500318. doi: 10.1002/cssc.202500318 (PMC12175033; doi:10.1002/cssc.202500318)
Supplement: Supplementary file 1 — Supplementary Material [file CSSC-18-e202500318-s001.pdf]

Supporting Information

N,N-dimethylhydrazone as a reversible derivatization agent to promote the hydroxymethylation of furfural with formaldehyde

Sarah Behloul,[a] Zhen Yan,[b] Karine De Oliveira Vigier,[a] Frederic Guegan[a] and François Jérôme.[a]*

1. General information 2
2. Analytical methods 3
   2.1 *NMR spectroscopy* 3
      2.1.1 Quantification-Measurement of relaxation times using the inversion recovery method 3
      2.1.2 Quantification-Exact content determination 6
   2.2 *Liquid chromatography-high resolution mass spectrometry (LC-HRMS) analysis* **Error! Bookmark not defined.**
3. Experimental procedure and chemical characterization of 2-(furan-2-ylmethylene)-1,1-dimethylhydrazine (**2**) 9
4. General analytical spectra 10
5. Impact of solvent on hydroxymethylation 15
6. Impact of sulfuric acid on hydroxymethylation 19
7. Impact of acidic catalysts on hydroxymethylation 25
8. Intermediate analytical spectra 26
9. HFIP-mediated hydroxymethylation 28
10. Guaiacol-mediated hydroxymethylation 31
11. Computational details 36
12. References 43

1. General information

Furfural was purified following the guidelines of Armarego and Chai prior to use.[1] The other reagents, catalyst and solvents were used as received from commercial suppliers (unless otherwise indicated).

Table S1. List, CAS number, suppliers and purity of reagents and catalysts used in this work

Name	CAS number	Purity	Supplier
1,1,1,3,3,3-Hexafluoro-2-propanol	920-66-1	>99%	Sigma-Aldrich
1,2-Ethanedithiol	540-63-6	98%	Sigma-Aldrich
1,3,5-Trimethoxybenzene	621-23-8	>99%	Sigma-Aldrich
2-Ethoxyphenol	94-71-3	98%	Sigma-Aldrich
2,2,2 - Trifluoroethanol	75-89-8	>99%	Sigma-Aldrich
3-Methyltrifluorophenol	98-17-9	99%	Sigma-Aldrich
Acetonitrile	75-05-8	>99.9	Fisher Scientific
Choline chloride	67-48-1	>98%	Sigma-Aldrich
Creosol	93-51-6	>98%	Sigma-Aldrich
Diglyme	111-96-6	99.5%	Sigma-Aldrich
Dimethylformamide	68-12-2	99.8%	Sigma-Aldrich
Ethanol	64-17-5	99%	Fisher Scientific
Ethylene glycol	107-21-1	>99%	Sigma-Aldrich
Formalin (37% formaldehyde in water)	50-00-0	37%	Thermo-Fisher Scientific
Furfural	98-01-1	97 %	Sigma-Aldrich
Glycerol	56-81-5	>99.5%	Sigma-Aldrich
Guaiacol	90-05-1	99%	Sigma-Aldrich
Isopropanol	67-63-0	99.5%	Fisher Scientific
Methanol	67-56-1	-	Fisher Scientific
N,*N*-dimethylhydrazine	57-14-7	98%	Sigma-Aldrich

Organic phases were concentrated under reduced pressure on a Heidolph rotary evaporator.

2. Analytical methods

2.1 NMR spectroscopy

^{1}H NMR and ^{13}C NMR spectra were recorded using a Bruker Ultrashield 500 Plus spectrometer operating at 500 MHz. All spectra were internally referenced to the residual proton signal of the solvent. Data for ^{1}H NMR are reported in terms of chemical shift (δ ppm), multiplicity (s = singlet, d = doublet, t = triplet, q = quartet, m = multiplet), coupling constants (Hz), and integration values.

Hydroxymethylation reactions were monitored *via* ^{1}H quantitative NMR analysis by diluting the reaction mixture in DMSO-*d*6 and adding a known mass of 1,3,5-trimethoxybenzene as an internal standard. The analysis focused on the spectral window of 5.9–7.5 ppm, where signals corresponding to the furan protons of the final alkylated and hydroxymethylated products were distinctly visible. The detailed experimental procedure is outlined below.

2.1.1 Quantification-Measurement of relaxation times using the inversion recovery method

The determination of longitudinal relaxation times T1 was conducted using the inversion recovery method at 25 °C. This experiment was carried out on a Bruker Ultrashield 500 plus (500MHz) spectrometer, employing the IconNMR software. The inversion recovery method is an automatic sequence available in the majority of spectrometers, including Bruker's IconNMR software.

The spectra of the inversion recovery method were acquired using the PROTONT1 sequence, with acquisition parameters adjusted based on the IconNMR Automation Interface manual available in the TopSpin software. The acquisition settings were as follows:

- Number of scan (ns): 2
- Dummy scan (DS): 0
- Acquisition time (AQ): 5.130s
- Relaxation delay (D1): 15s
- Pulse length (P1): 90°
- Pulse length (P2): 180°
- The following vdlist has been established for proton T1 measurement (times are listed in seconds): 0.05, 0.10, 0.15, 0.20, 0.30, 0.35, 0.50, 0.70, 1.00, 1.40, 2.00, 3.00, 4.50, 7.00, 10.00, 15.00, 25.00, 35.00 and 40.00 (and extended to 45.00, 50.00, 55.00, 60.00, 65.00, 70.00 in case of furfural).

Processing and data analysis were automatically performed in TopSpin according to the standard procedure to determine the different parameters of Eq.1, including the relaxation time T1.

[1]H inversion-recovery curves of the different studied compounds are given as follow.

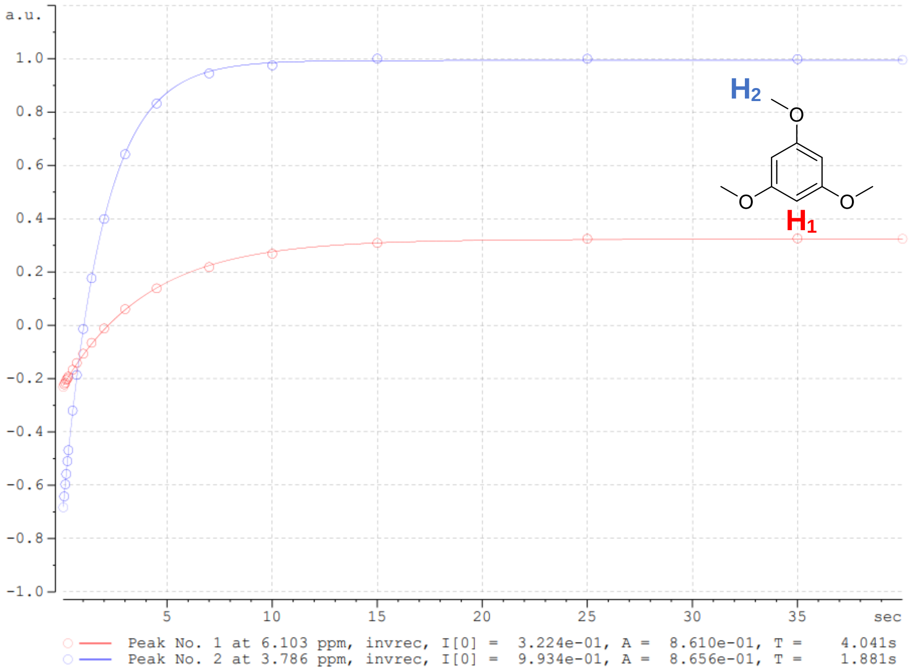

Figure S1: Relative intensity of the recovery longitudinal magnetization (Mz) as a function of time (vd) of 1,3,5-trimethoxybenzene.

The inversion-recovery experiment displayed was acquired using IconNMR.
Proton relaxation times in $CDCl_3$ are given as follow: 4.041s H_1 and 1.881s H_2.

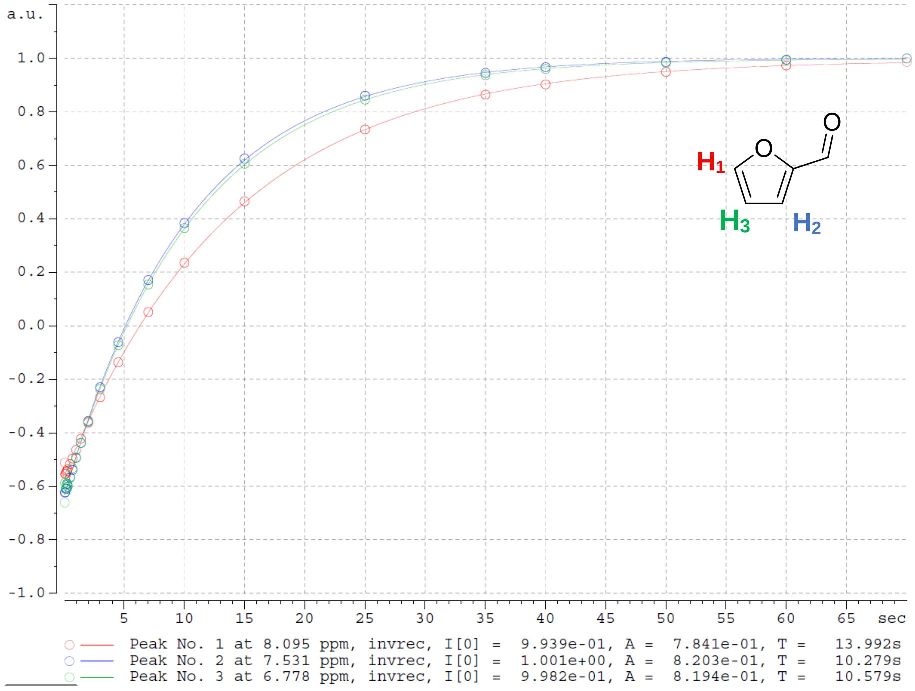

Figure S2: Relative intensity of the recovery longitudinal magnetization (Mz) as a function of time (vd) of furfural.

The inversion-recovery experiment displayed was acquired using IconNMR.
Proton relaxation times in DMSO-d6 are given as follow: 13.992 H_1, 10.279 H_2, 10.579s H_3.

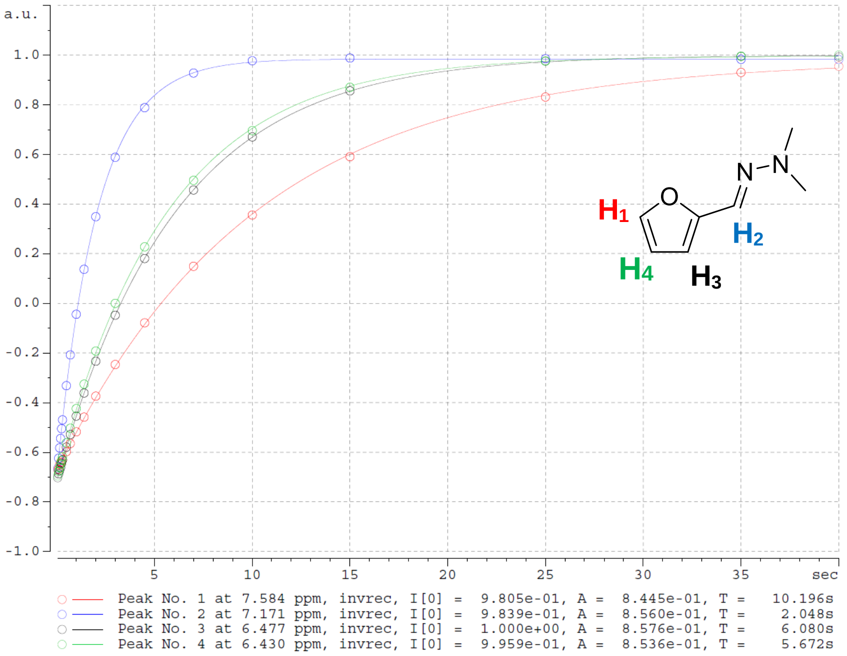

Figure S3: Relative intensity of the recovery longitudinal magnetization (Mz) as a function of time (vd) of of 2-(furan-2-ylmethylene)-1,1-dimethylhydrazine (**2**).

The inversion-recovery experiment displayed was acquired using IconNMR.
Proton relaxation times in DMSO-d6 are given as follow: 10.196s H_1, 2.048s H_2, 6.080s H_3 and 5.672s H_4.

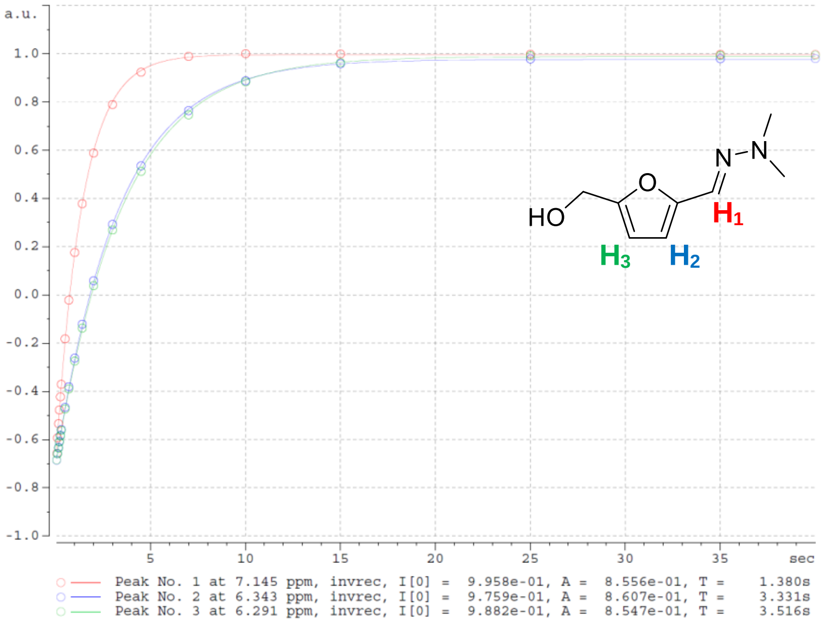

Figure S4: Relative intensity of the recovery longitudinal magnetization (Mz) as a function of time (vd) of (5-((2,2-dimethylhydrazineylidene)methyl)furan-2-yl)methanol (**3**).

The inversion-recovery experiment displayed was acquired using IconNMR.

Proton relaxation times in DMSO-*d*6 are given as follow: 1.380 H_1, 3.331 H_2, 3.516s H_3.

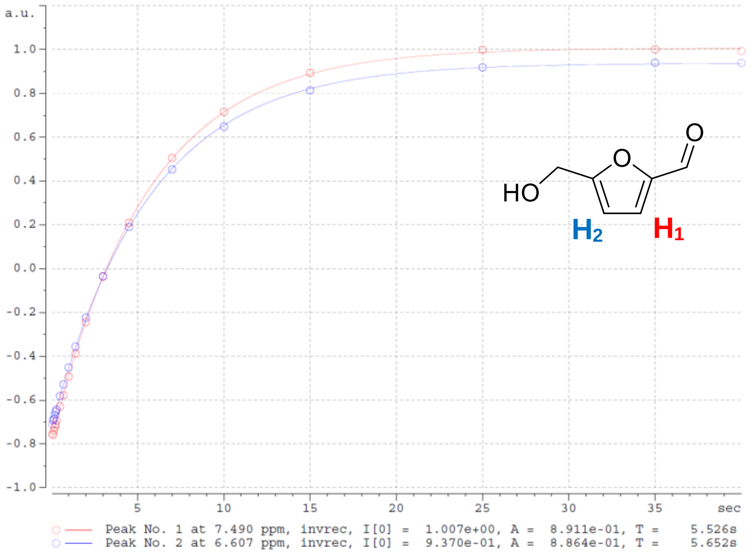

Figure S5: Relative intensity of the recovery longitudinal magnetization (Mz) as a function of time (vd) of HMF.

The inversion-recovery experiment displayed was acquired using IconNMR.
Proton relaxation times in DMSO-d6 are given as follow: 5.526s H_1 and 5.652s H_2.

2.1.2 Quantification-Exact content determination

Quantitative NMR was employed to determine the initial purity of reagents and monitor reaction progress. Approximately 10 mg of 1,3,5-trimethoxybenzene (internal standard) and 25 mg of the crude reaction sample were accurately weighed, dissolved in 0.7 mL of DMSO-d6, and transferred to an NMR tube. Acquisition parameters were optimized for accurate quantification, with a relaxation delay set to five times the longest T1 value of the protons analyzed.

Purity (P_X) of a compound X was calculated using the following equation [4]:

$$P_X = \frac{I_X}{I_S} \times \frac{N_S}{N_X} \times \frac{M_X}{M_S} \times \frac{w_S}{w_C} \times P_S$$

Where I, N, M, w and P denote the integrated signal area, number of protons, molecular mass, weighed mass, and purity (as a mass fraction) for the compound X and the internal standard S.

The exact mass of X in the crude reaction mixture (w_X) was determined using:

$$w_X = P_X \times \ w_{TC}$$

The moles of X were then calculated as:

$$n_X = \frac{w_X}{M_X}$$

Yield, conversion, and selectivity were subsequently determined using:

$$Yield\ (\%) = \frac{(n_X/a)}{(\frac{n_{R,0}}{b})}$$

$$Conversion\ (\%) = \frac{n_{R,0} - n_{R,t}}{n_{R,0}} \times 100$$

$$Selectivity\ (\%) = \frac{Yield\ (\%)}{Conversion\ (\%)} \times 100$$

Where $n_{R,0}$ is the initial molar quantity of the reactant, $n_{R,t}$ the molar quantity of the reactant at a time t, a and b the stoichiometric coefficient of X and R respectively.

In cases of phase immiscibility, absolute ethanol was added to achieve a clear single phase, and dilution was accounted for during quantification.

2.2 Ultra-High Performance Liquid Chromatography-High Resolution Mass Spectrometry (UHPLC-HRMS)

LC-HRMS analyses were carried out using a Thermo scientific Ultimate 3000 system coupled with a quadrupole-Orbitrap (Q Exactive) mass spectrometer equipped with a HESI source. Chromatographic separation was performed on a Kinetex F5 100 Å column (150 x 2.1 mm, 1.7 µm) with a mobile phase composed of ultra-pure water and acetonitrile, both containing 0.1% of formic acid, at a flow rate of 0.4 mL/min. After injection of 10 µL of sample, the gradient was programmed as presented in Table S2.

Table S2. Program used for HPLC analysis.

Time (min)	%ultrapure water	%acetonitrile
0	1.0	99.0
0.5	1.0	99.0
2.8	10.0	90.0
14.8	99.0	1.0
15.8	99.0	1.0
16.8	1.0	99.0
19.0	1.0	99.0

The HESI source was operated with a sheath gas flow rate of 40, an auxiliary gas flow rate of 30, and a sweep gas flow rate of 0. The spray voltage was set to 2.80 kV, the capillary temperature was maintained at 150°C, and the auxiliary gas heater temperature was set to 300°C.

Mass error (in ppm) was calculated as follow [5]:

$$Mass\ error\ (ppm) = \frac{Observed\ m.z - Theoretical\ m.z}{Theoretical\ m.z} \times 10^6$$

3. Experimental procedure and chemical characterization of 2-(furan-2-ylmethylene)-1,1-dimethylhydrazine (**2**)

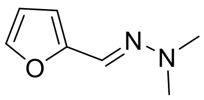

2-(furan-2-ylmethylene)-1,1-dimethylhydrazine was synthetized according to a procedure previously described by Kamimura and coworkers.[6] 0.69 mL (8 mmol) of freshly distilled furfural, 0.82 mL of *N*,*N*-dimethylhydrazine (10 mmol) and 60 mL of ethanol were charged in a single-neck round bottom flask and stirred at 25 °C for 2 h. The reaction is quantitative. The reaction mixture was concentrated using a rotary evaporator to remove EtOH. The remaining aqueous mixture was extracted with ethyl acetate (3 × 30 mL). The organic layer was dried ($MgSO_4$), filtered and concentrated in vacuo to afford the product as a yellow liquid (1.06 g, 91%).

1H NMR (500 MHz, $CDCl_3$) δ 7.36 (dd, $^3J_{H-H}$ =1.7 Hz, $^4J_{H-H}$ =0.5 Hz, 1H, =*CH*), 7.11 (s, 1H, -*C(H)=N),* 6.38 (dd, $^3J_{H-H}$ = 3.3 Hz, 1.7 Hz, 1H, =*CH*), 6.34 (d, $^3J_{H-H}$ = 3.3 Hz, 1H, =*CH*), 2.94 (s, 6H, -*N(CH₃)₂*).

13C NMR (125 MHz, $CDCl_3$) δ 152.09, 141.98, 123.39, 111.31, 107.35, 42.85.

GC-HRMS: m/z calculated for $C_7H_{10}N_2O$ 138.0793, found 138.0794 (mass error: 0.72 ppm).

4. General analytical spectra

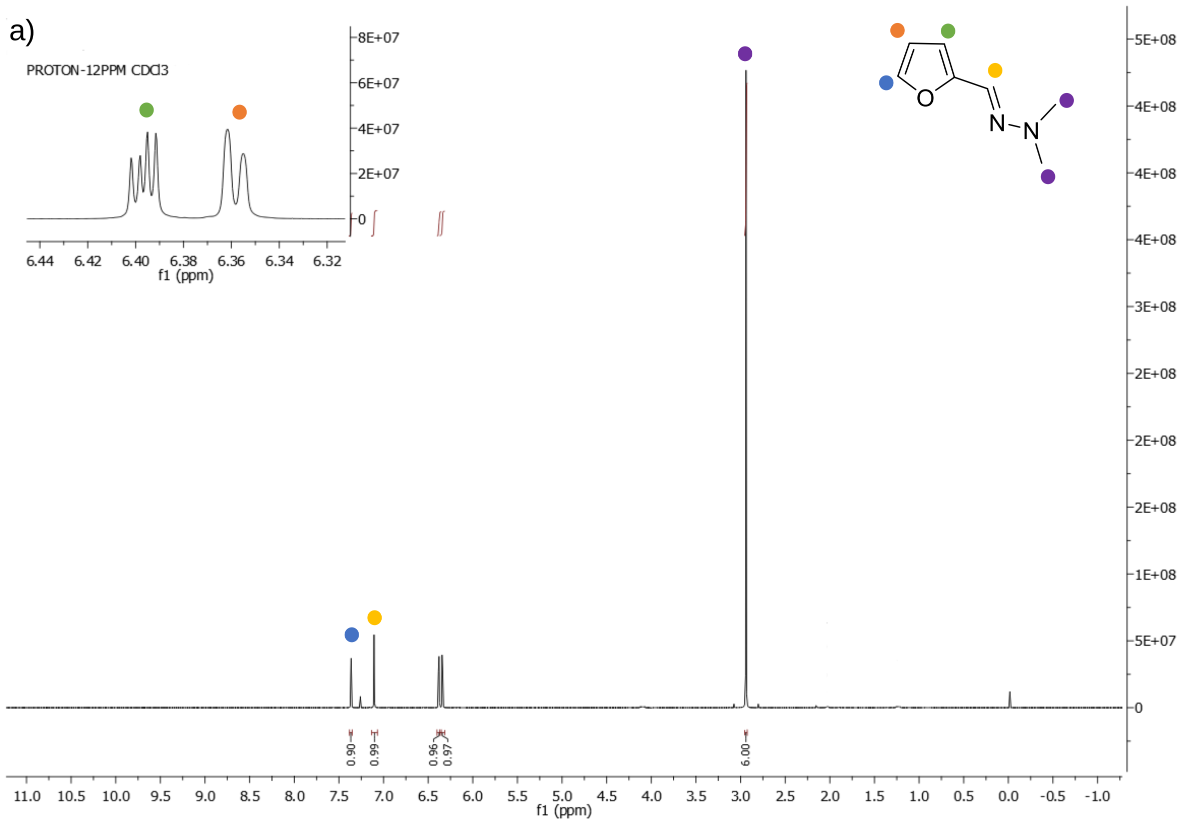

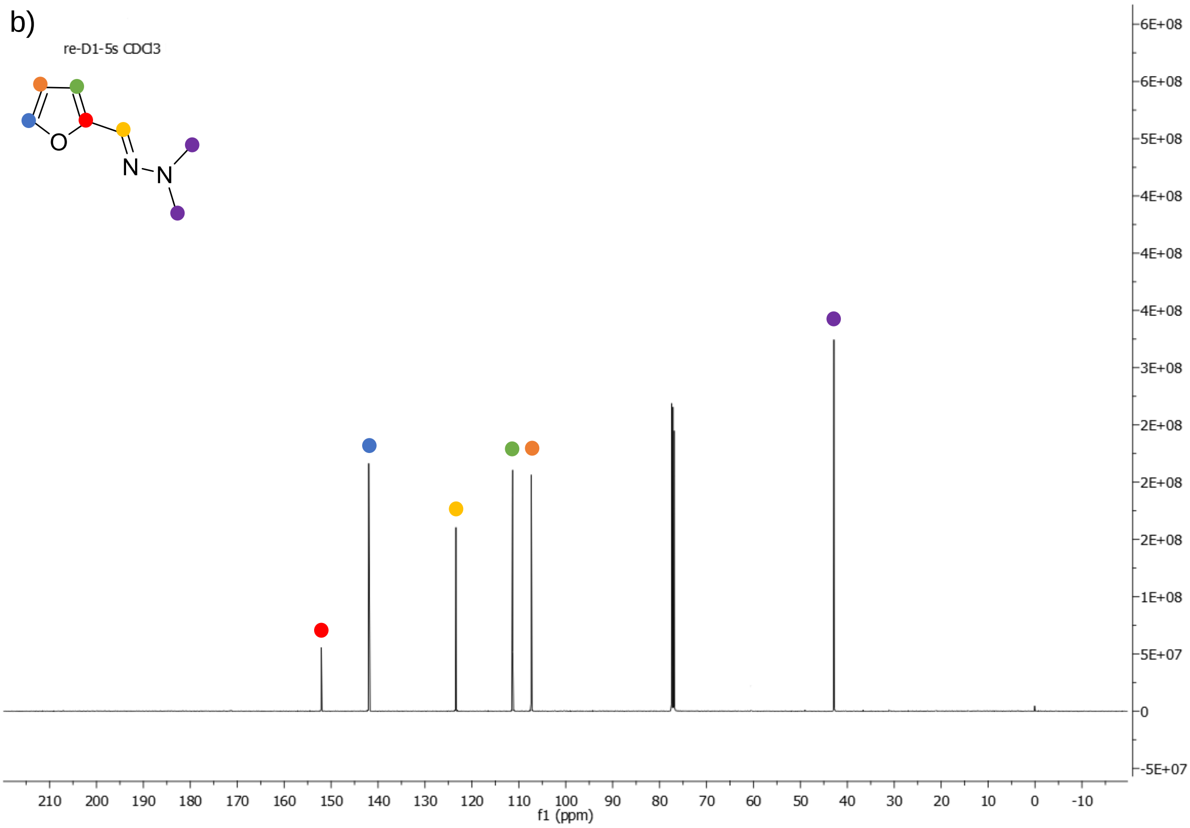

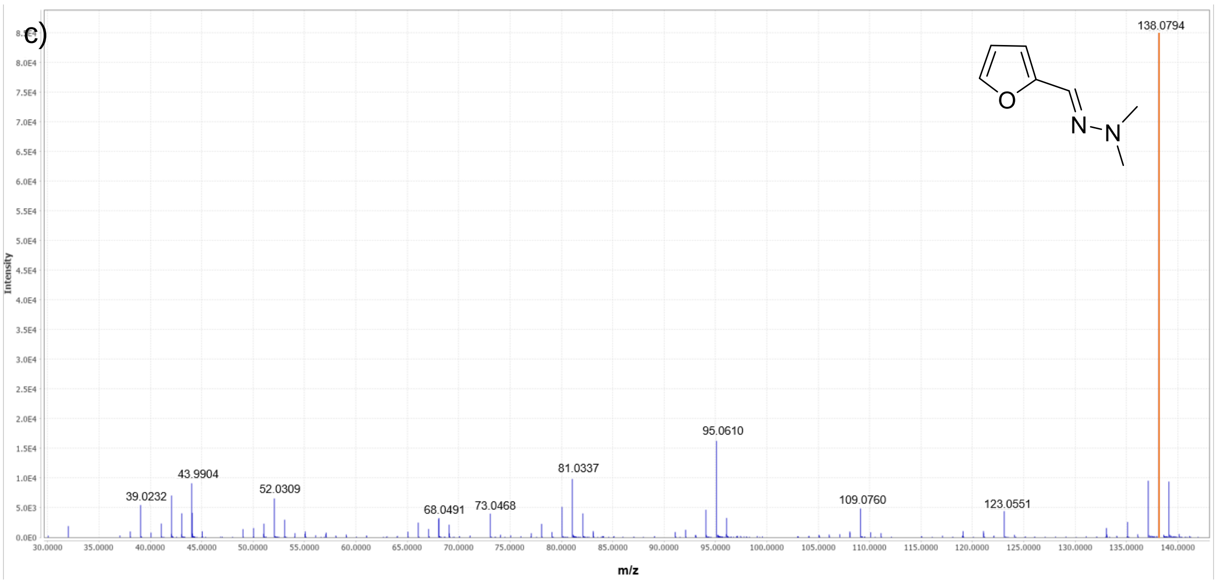

Figure S6: NMR spectra of 2-(furan-2-ylmethylene)-1,1-dimethylhydrazine (**2**) (a- ^{1}H spectrum, b- ^{13}C spectrum) and mass spectrum (c).

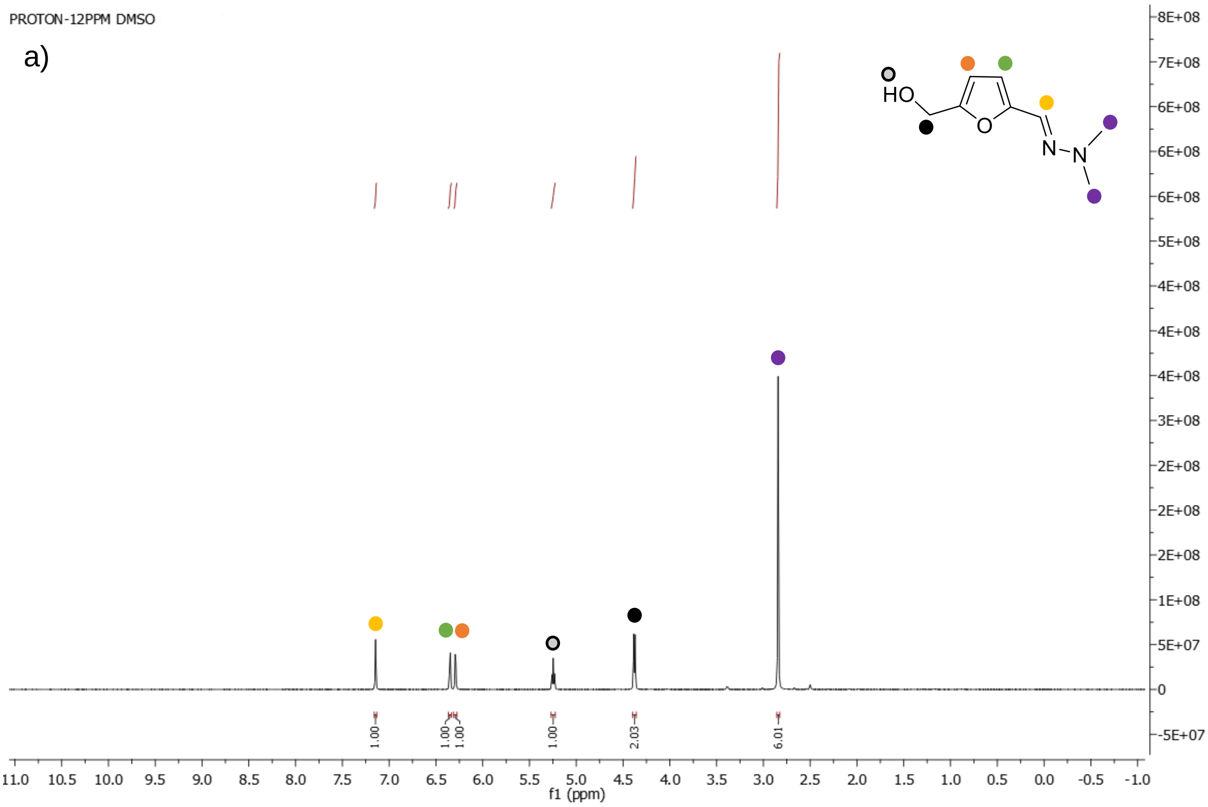

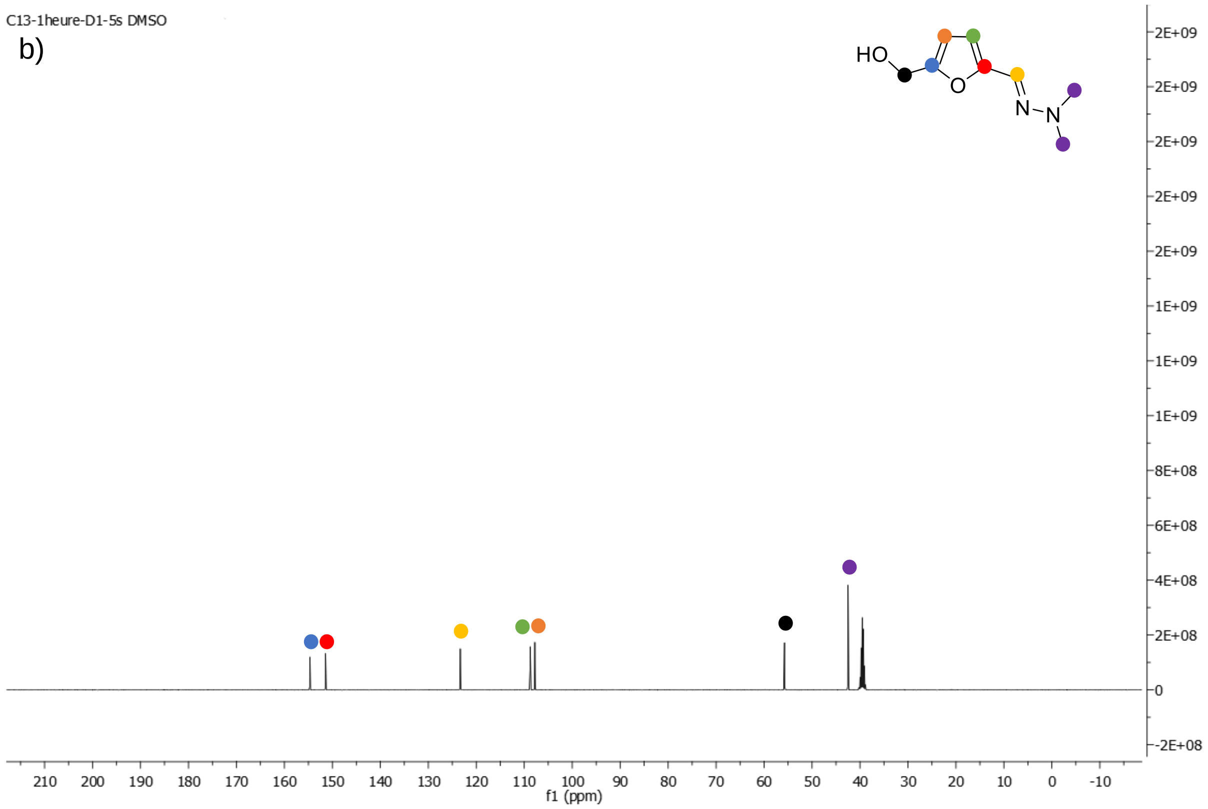

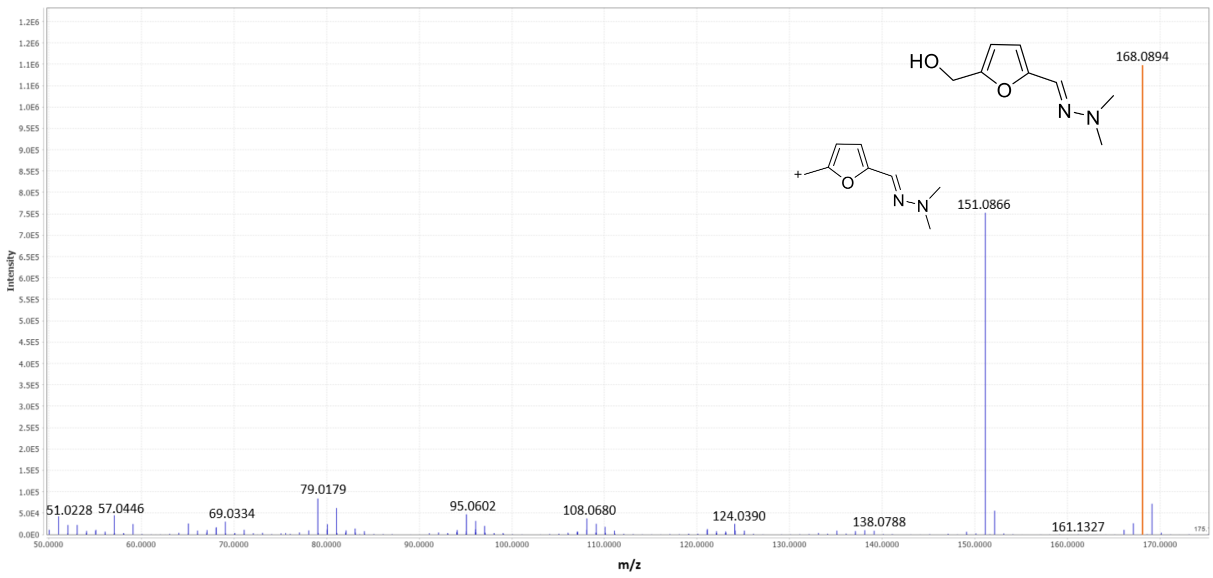

Figure S7: NMR spectra of of (5-((2,2-dimethylhydrazineylidene)methyl)furan-2-yl)methanol (**3**) (a- ^{1}H spectrum, b- ^{13}C spectrum) and mass spectrum including a hypothetic associated fragment (c) – calculated on Mass Frontier™ 8.0.

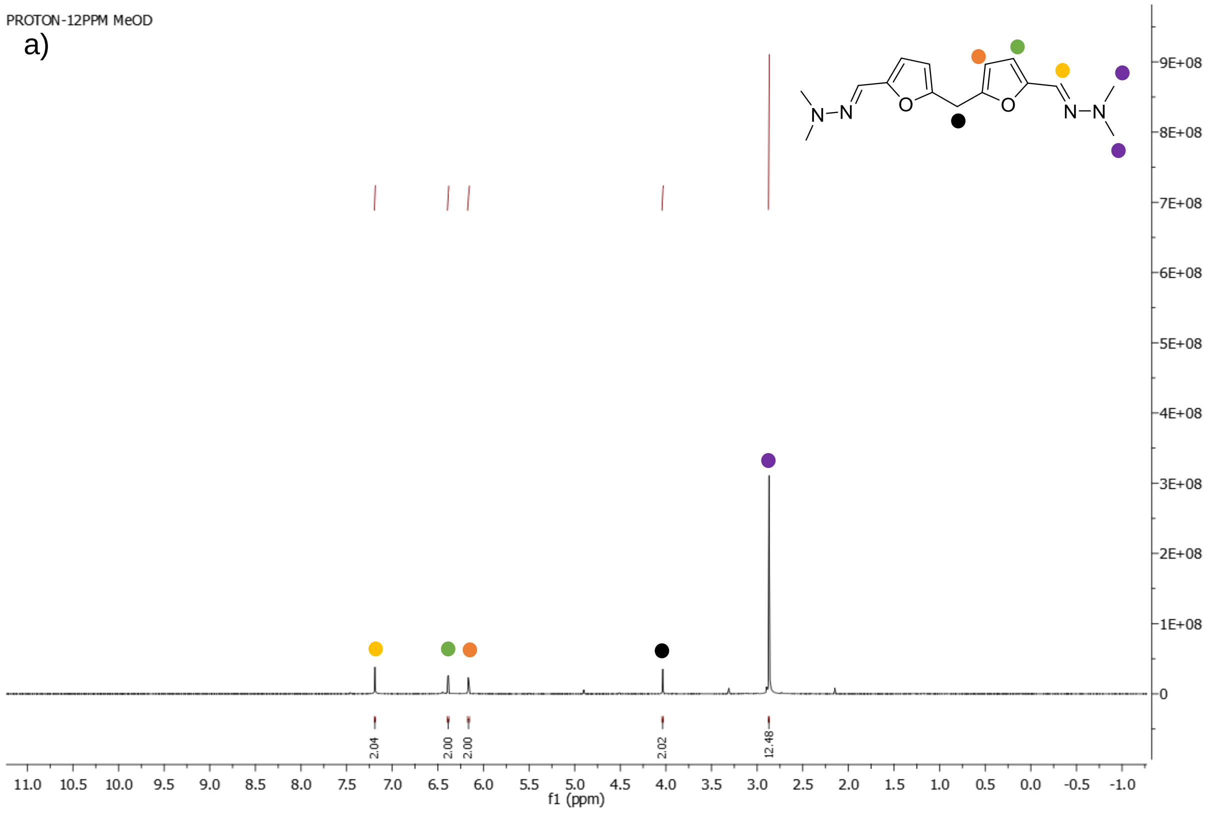
PROTON-12PPM MeOD
a)
N–N
O
O
N–N
2.04
2.00
2.00
2.02
12.48
f1 (ppm)

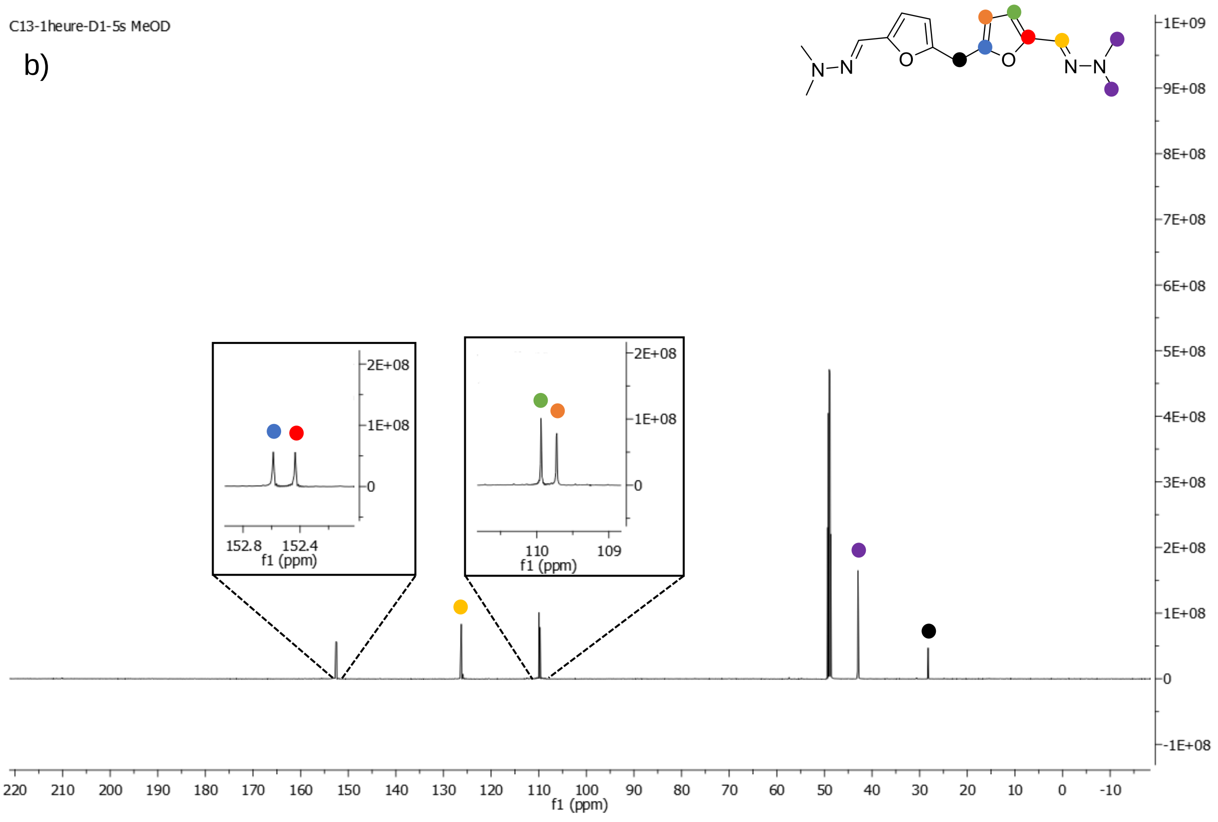
C13-1heure-D1-5s MeOD
b)
N–N
O
O
N–N
152.8
152.4
f1 (ppm)
110
109
f1 (ppm)
f1 (ppm)

c)

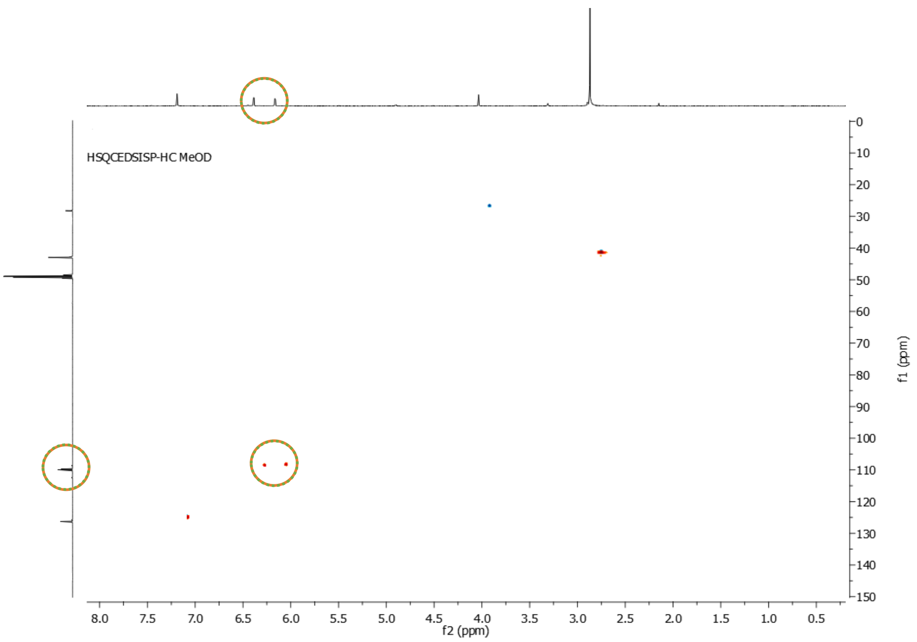

d)

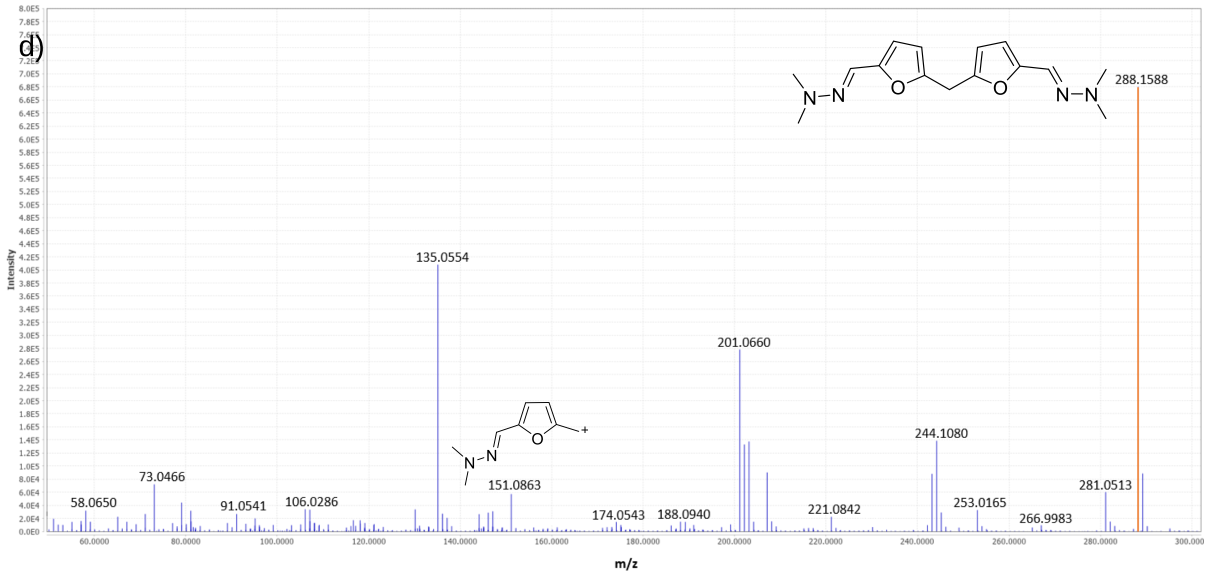

Figure S8: NMR spectra of bis(5-(2,2-dimethylhydrazineylidene)methyl)furan-2-yl)methane (**4**) (a- ^{1}H spectrum, b- ^{13}C spectrum, c-HSQC) and mass spectrum including a hypothetic associated fragment (d) – calculated on Mass Frontier^{TM} 8.0.

5. Impact of solvent on hydroxymethylation

In this study, each kinetic data point was obtained from an independent reaction (one data point per experiment) to eliminate potential sampling effects. This approach was made possible by the straightforward and scalable synthesis of furfural dimethylhydrazone (**2**), which was pure enough for direct use without the need for further purification.

As previously noted, when the reaction medium exhibited heterogeneity, a minimal amount of absolute ethanol was introduced to ensure the formation of a homogeneous, single-phase system. For each experiment, two samples were collected, and two separate quantitative NMR analyses were performed to ensure reliability.

Reaction conditions: in a 2-mL round-bottom flask, furfural dimethylhydrazone **2** (1 eq., 11 wt.%), formaldehyde (37% water, 1 eq.) and a solvent were stirred at 50 °C.

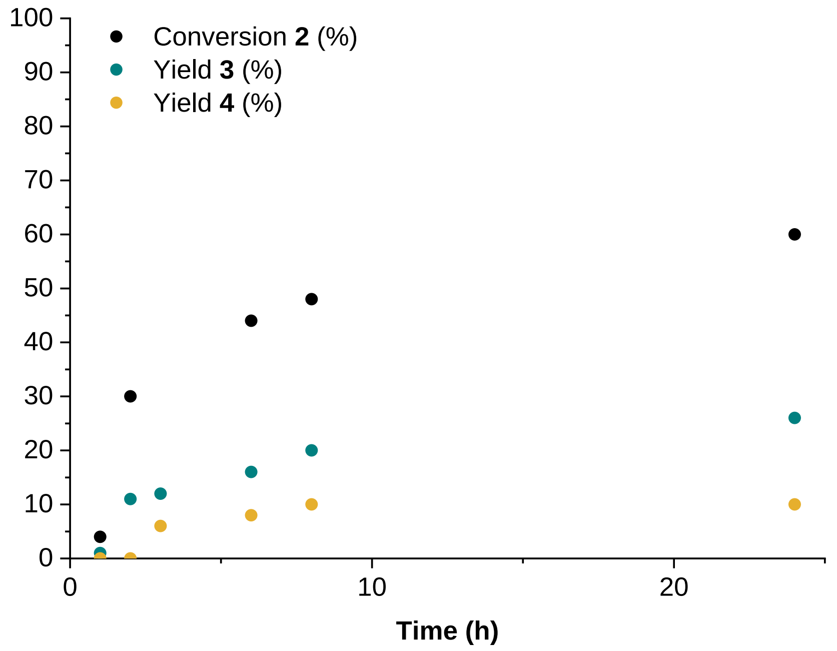

Figure S9: Conversion (**2**) and yields (**3, 4**) in hydroxymethylation without any solvent.

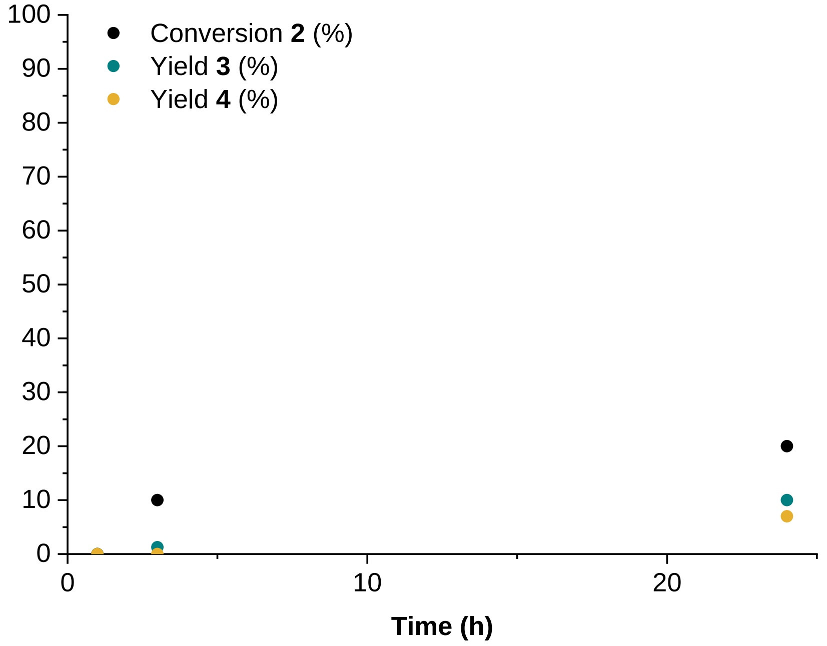

Figure S10: Conversion (**2**) and yields (**3, 4**) in hydroxymethylation using ethylene glycol.

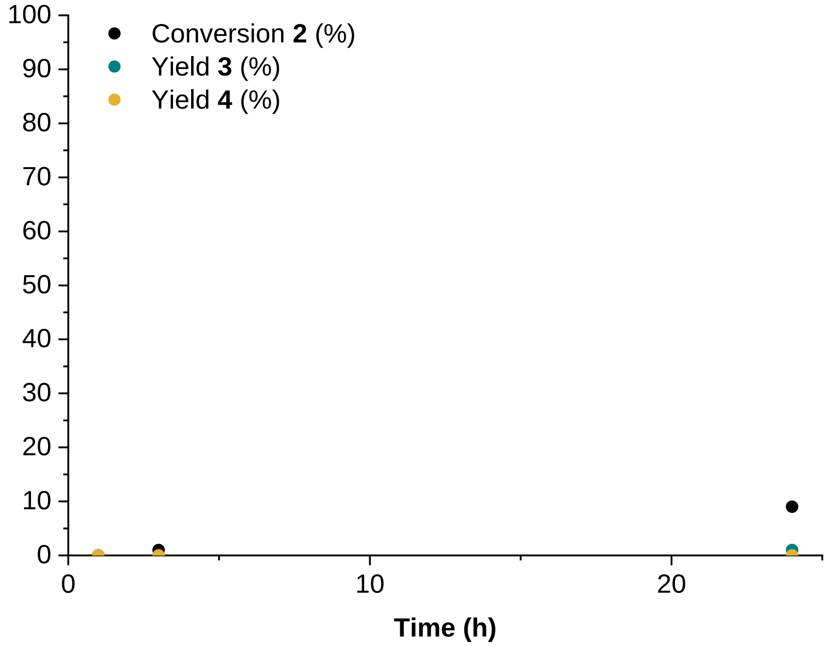

Figure S11: Conversion (**2**) and yields (**3, 4**) in hydroxymethylation using methanol.

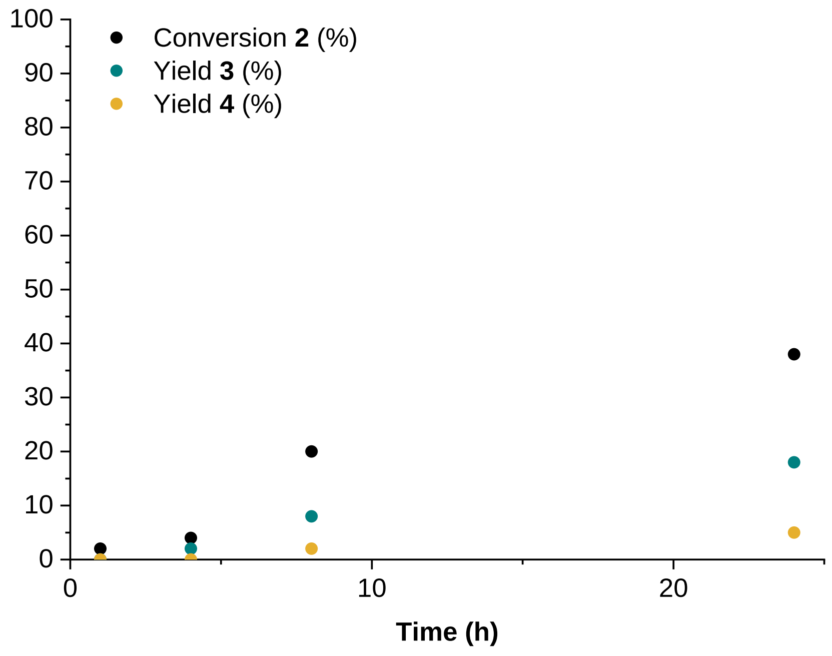

Figure S12: Conversion (**2**) and yields (**3, 4**) in hydroxymethylation using water / methanol.

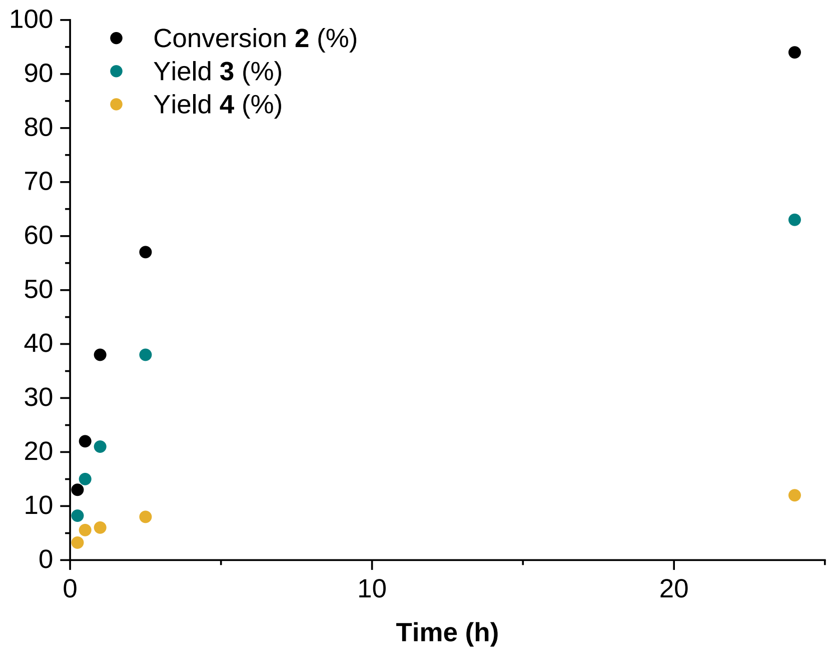

Figure S13: Conversion (**2**) and yields (**3, 4**) in hydroxymethylation using trifluoroethanol.

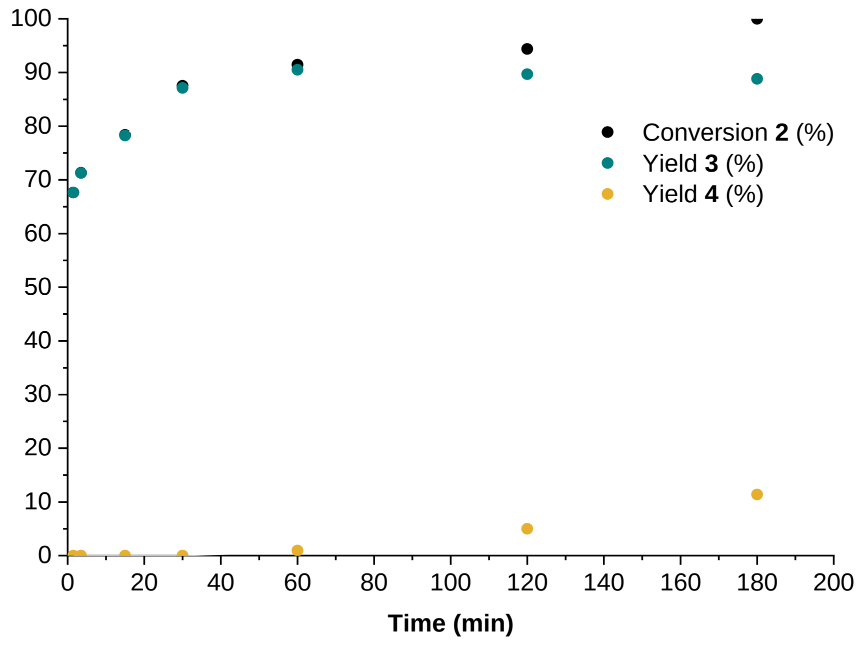

Figure S14: Conversion (**2**) and yields (**3, 4**) in hydroxymethylation using HFIP.

6. Impact of sulfuric acid on hydroxymethylation

In this study, each kinetic data point was obtained from an independent reaction (one data point per experiment) to eliminate potential sampling effects. This approach was made possible by the straightforward and scalable synthesis of furfural dimethylhydrazone (**2**), which was pure enough for direct use without the need for further purification.

As previously noted, when the reaction medium exhibited heterogeneity, a minimal amount of absolute ethanol was introduced to ensure the formation of a homogeneous, single-phase system. For each experiment, two samples were collected, and two separate quantitative NMR analyses were performed to ensure reliability.

Reaction conditions: in a 2-mL round-bottom flask, furfural dimethylhydrazone **2** (1 eq., 11 wt.%), formaldehyde (37% water, 1 eq.), sulfuric acid (0.1 eq.) and a solvent were stirred at 50 °C.

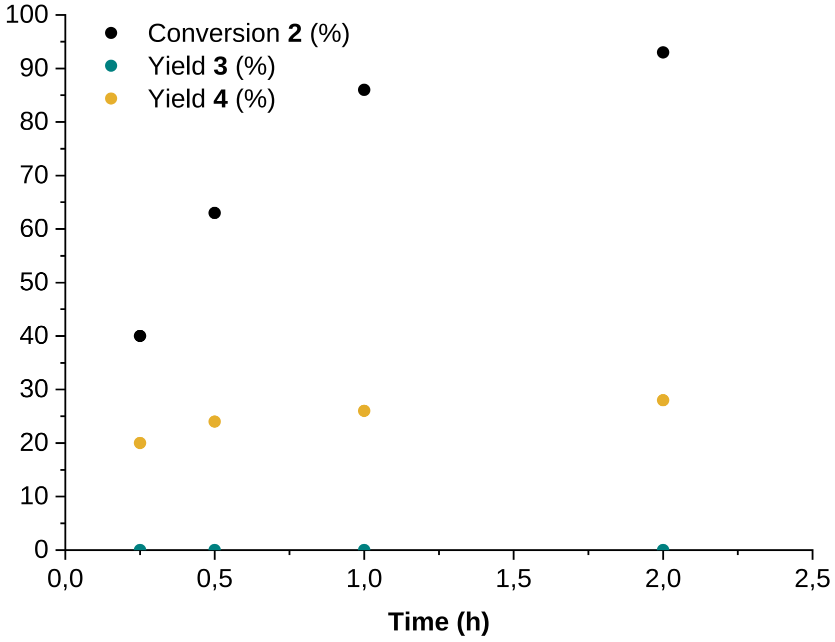

Figure S15: Conversion (**2**) and yields (**3, 4**) in hydroxymethylation without any solvent.

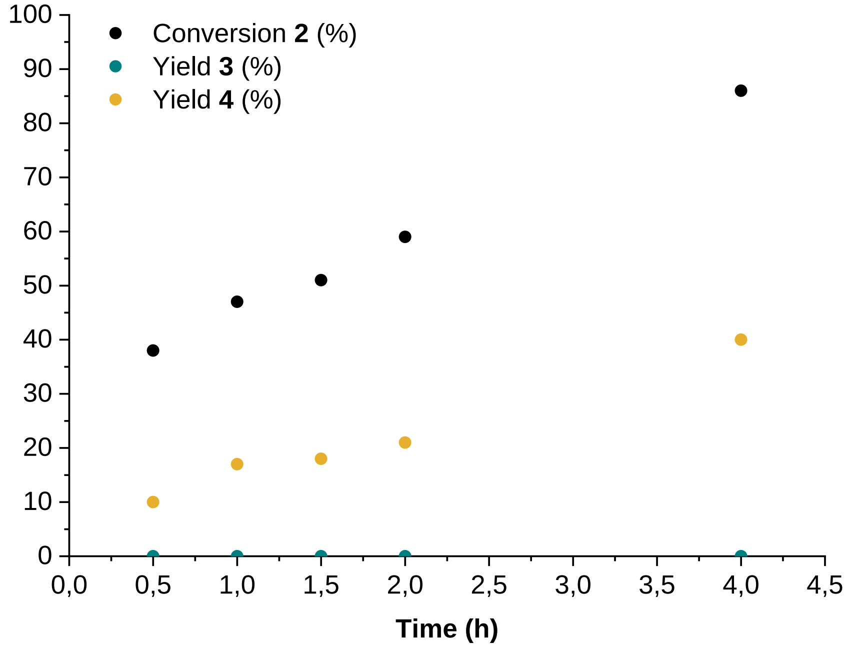

Figure S16: Conversion (**2**) and yields (**3, 4**) in hydroxymethylation with glycerol.

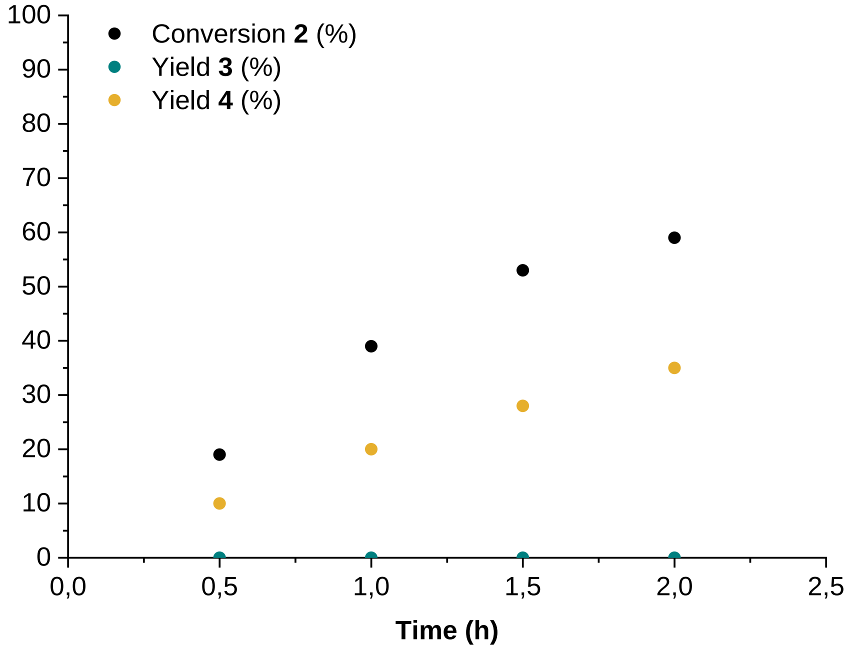

Figure S17: Conversion (**2**) and yields (**3, 4**) in hydroxymethylation with ethylene glycol.

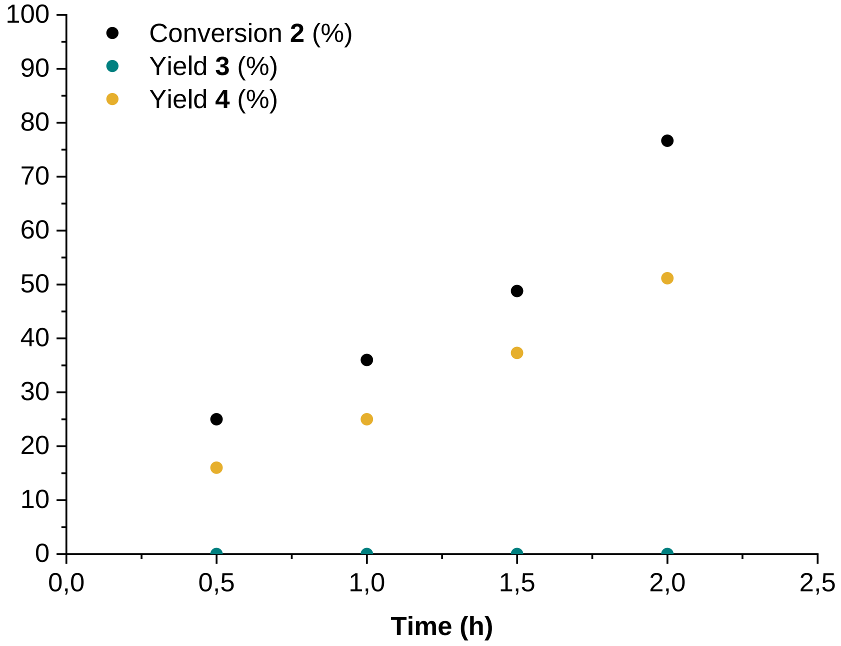

Figure S18: Conversion (**2**) and yields (**3, 4**) in hydroxymethylation with methanol.

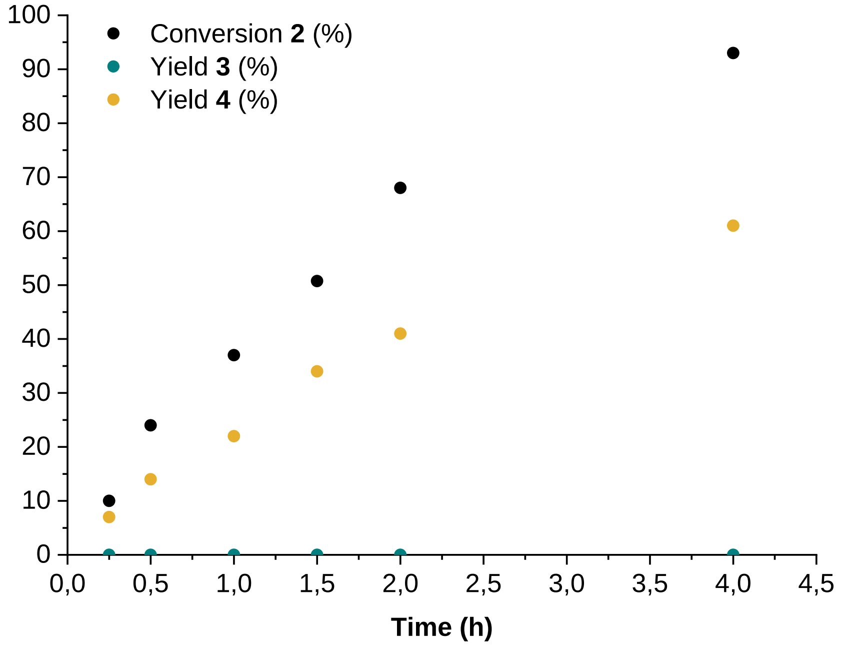

Figure S19: Conversion (**2**) and yields (**3, 4**) in hydroxymethylation with ethanol.

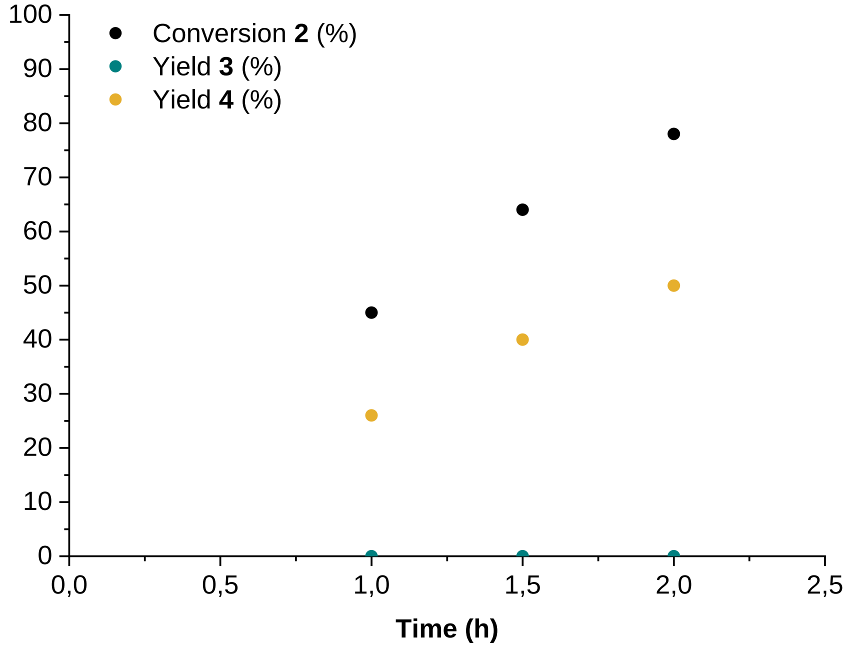

Figure S20: Conversion (**2**) and yields (**3, 4**) in hydroxymethylation with isopropanol.

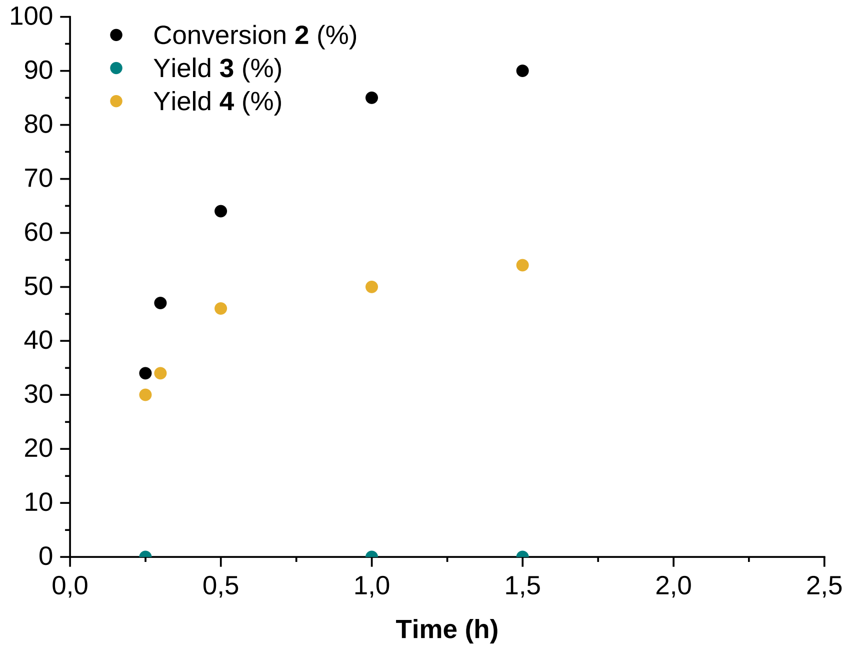

Figure S21: Conversion (**2**) and yields (**3, 4**) in hydroxymethylation with acetonitrile.

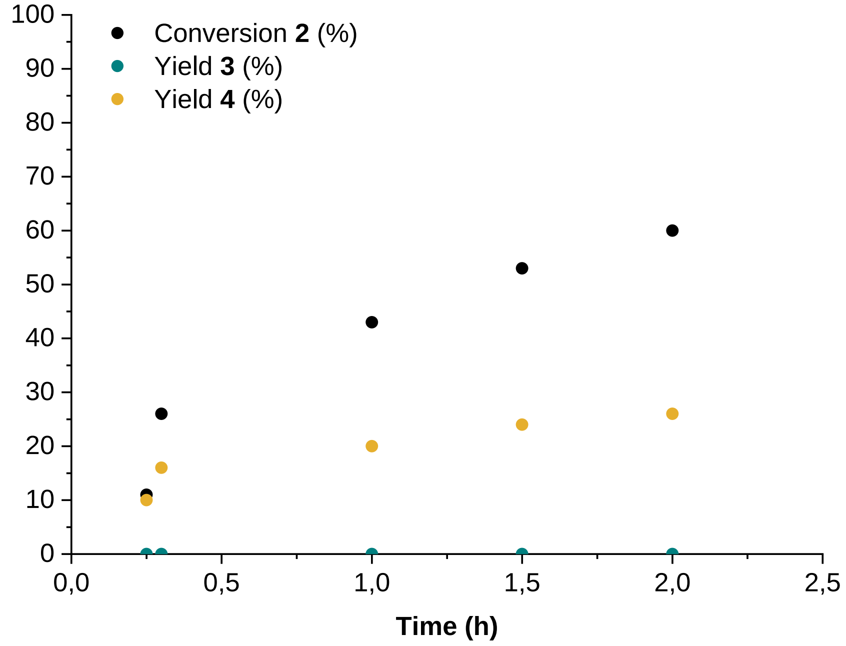

Figure S22: Conversion (**2**) and yields (**3, 4**) in hydroxymethylation with dimethylformamide.

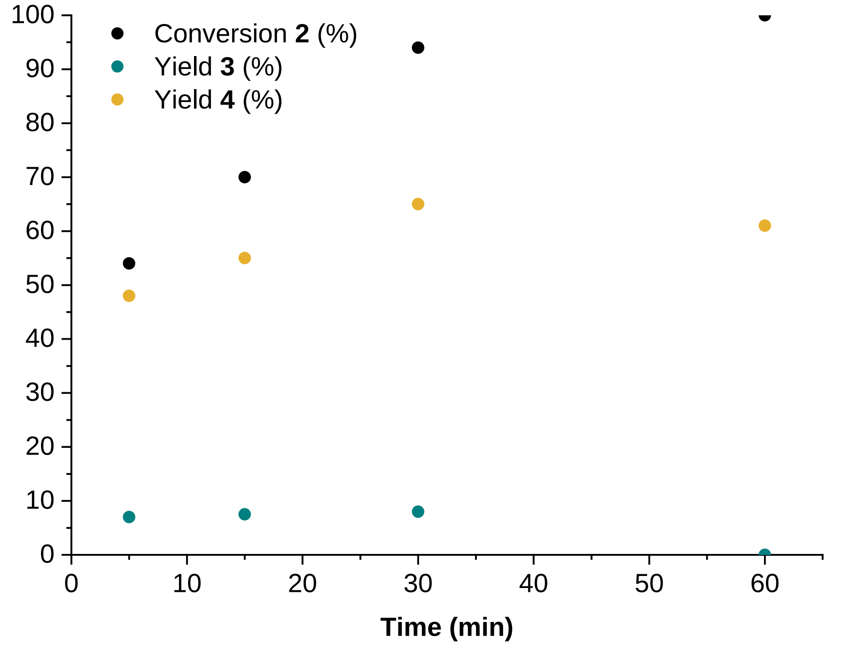

Figure S23: Conversion (**2**) and yields (**3, 4**) in hydroxymethylation with trifluoroethanol.

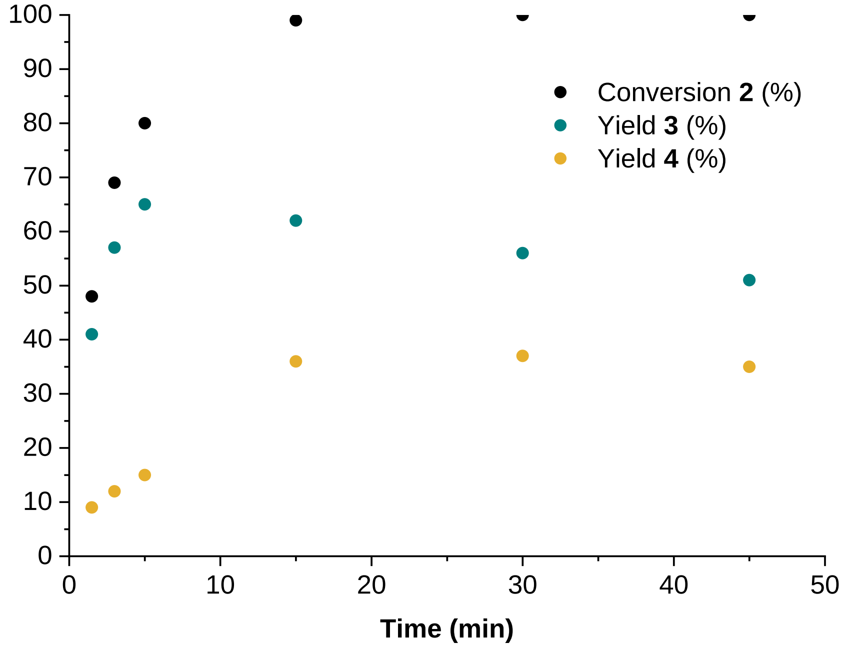

Figure S24: Conversion (**2**) and yields (**3, 4**) in hydroxymethylation with HFIP.

7. Impact of acidic catalysts on hydroxymethylation

Reaction conditions: in a 2-mL round-bottom flask, furfural dimethylhydrazone **2** (1 eq., 11 wt.%), formaldehyde (37% water, 1 eq.), an acid catalyst and a solvent were stirred at 50 °C otherwise indicated.

Table S3. Conversion and yields of hydroxymethylation using various acidic conditions.

Entry	Catalyst	Solvent	Conversion 2 (%) (time)	Yield 3 (%) (time)	Yield 4 (%) (time)
1	Acetic acid	Ethylene glycol	82 (24 h)	0 (24 h)	75 (24 h)
2[a]	Formic acid	Ethylene glycol	26 (20 h)	0 (20 h)	24 (20 h)
3	Trifluoroacetic acid	Ethanol	100 (24 h)	0 (24 h)	58 (24 h)
4	Trifluoroacetic acid	None	60 (15 min)	0 (15 min)	12 (15 min)
5[a]	Betaine hydrochloride	Ethanol	38 (24 h)	0 (24 h)	26 (24 h)
6[a]	Formic acid	Ethanol	26 (24 h)	0 (24 h)	23 (24 h)
7	Aquivion PW98	THF	51 (2 h)<br>85 (20 h)	0 (2 h)<br>0 (20 h)	50 (2 h)<br>68 (20 h)
8	Bismuth(III) triflate	Ethanol	100 (24 h)	0 (24 h)	72 (24 h)
9	Hydrochloric acid	Ethanol	95 (24 h)	0 (24 h)	70 (24 h)
10	Aluminium chloride	Ethanol	98 (24 h)	0 (24 h)	68 (24 h)

[a] Experiment performed at 25 °C.

8. Intermediate analytical spectra

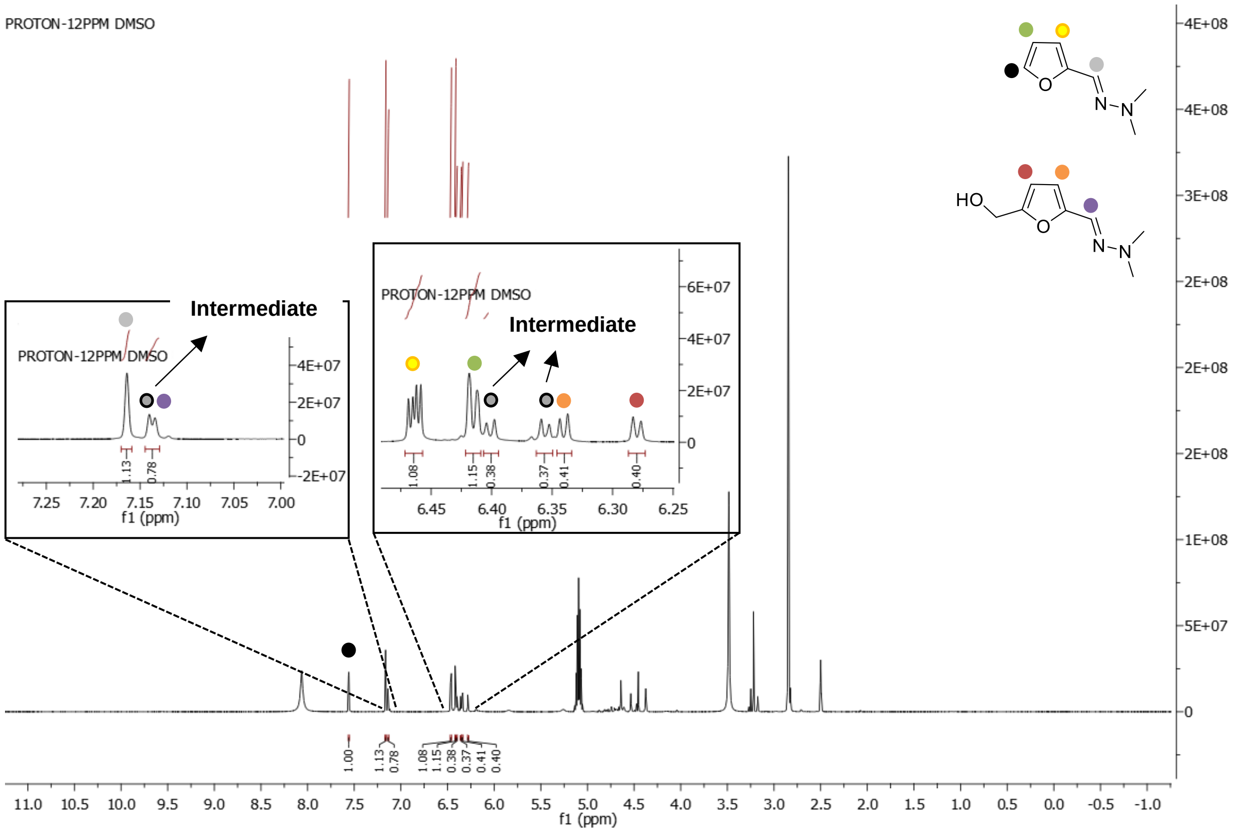

Figure S25: Resulting NMR spectra of the crude of (**2**) and formaldehyde in HFIP (3 eq.) at 50 °C after 2 min (grey and black circles: intermediate signals).

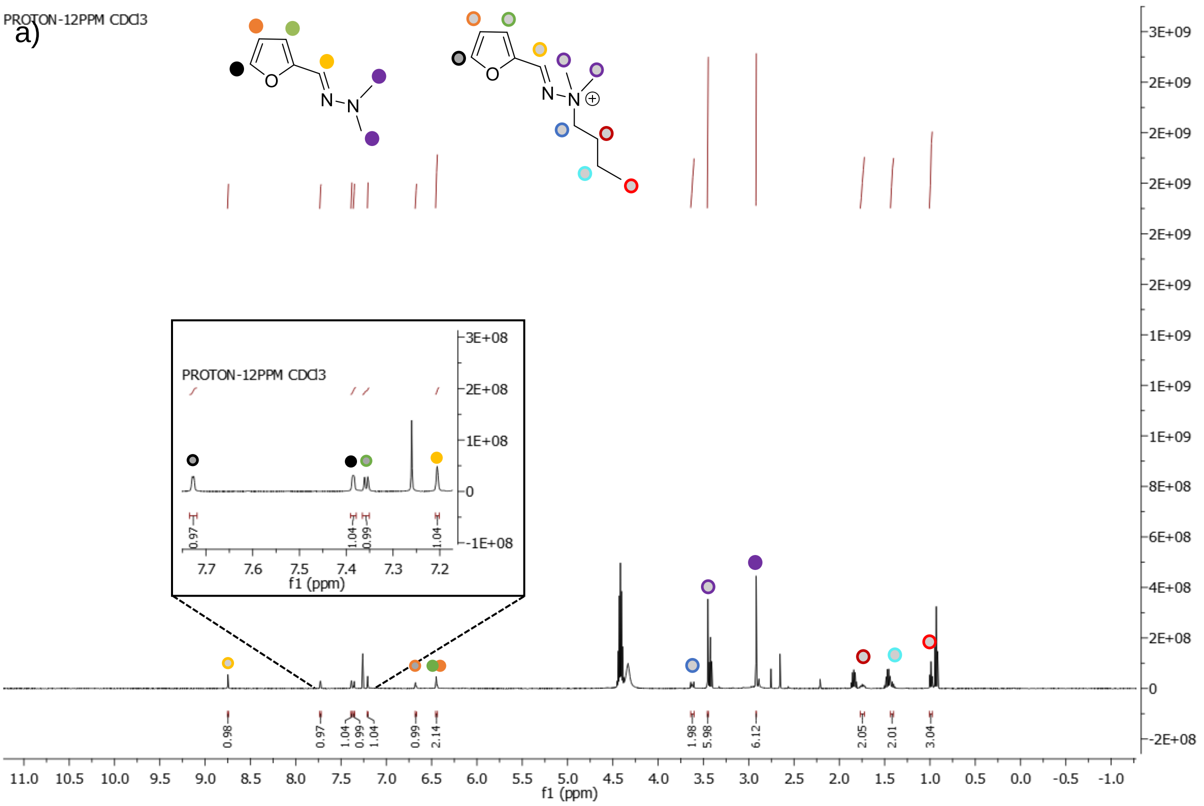

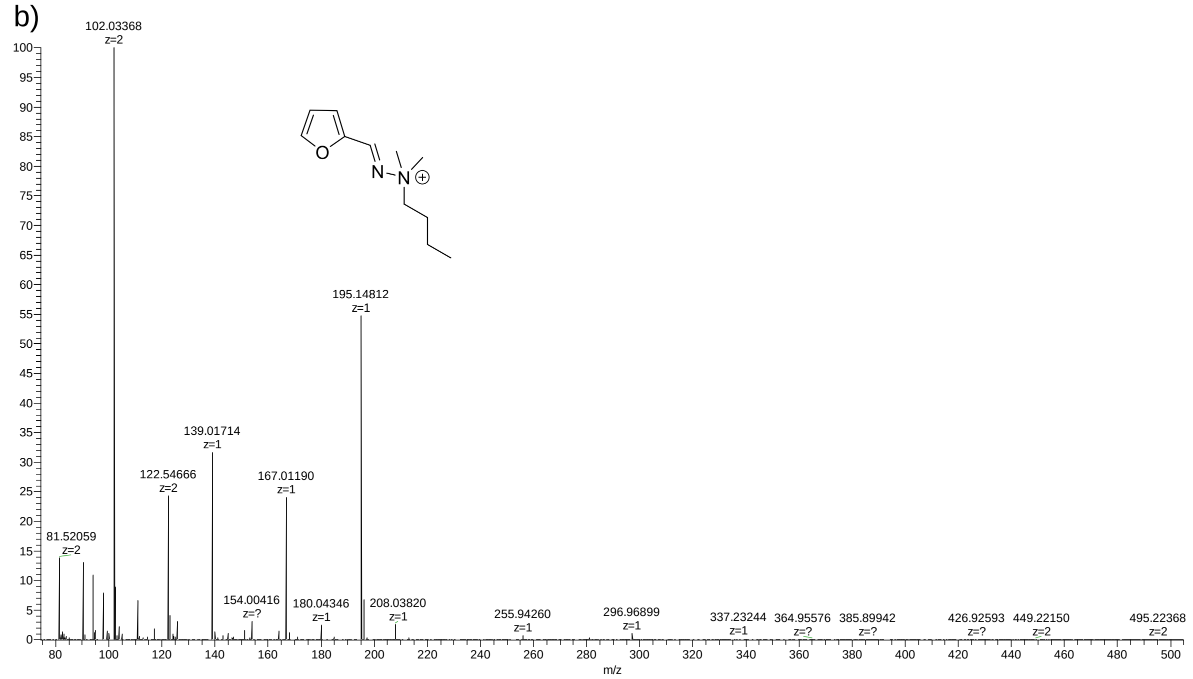

Figure S26: Resulting NMR (a) and mass (b)spectra of the crude of (**2**) and *n*-bromobutane in HFIP (11 eq.) at 50 °C after 24 hours.

9. HFIP-mediated hydroxymethylation

In the kinetic study, each data point was obtained from an independent reaction (one data point per experiment) to eliminate potential sampling effects. This approach was made possible by the straightforward and scalable synthesis of furfural dimethylhydrazone (**2**), which was pure enough for direct use without the need for further purification.

As previously noted, when the reaction medium exhibited heterogeneity, a minimal amount of absolute ethanol was introduced to ensure the formation of a homogeneous, single-phase system. For each experiment, two samples were collected, and two separate quantitative NMR analyses were performed to ensure reliability.

Reaction conditions: in a 2-mL round-bottom flask, furfural dimethylhydrazone **2** (1 eq.), formaldehyde (37% water, 1 eq.) and HFIP were stirred at 50 °C.

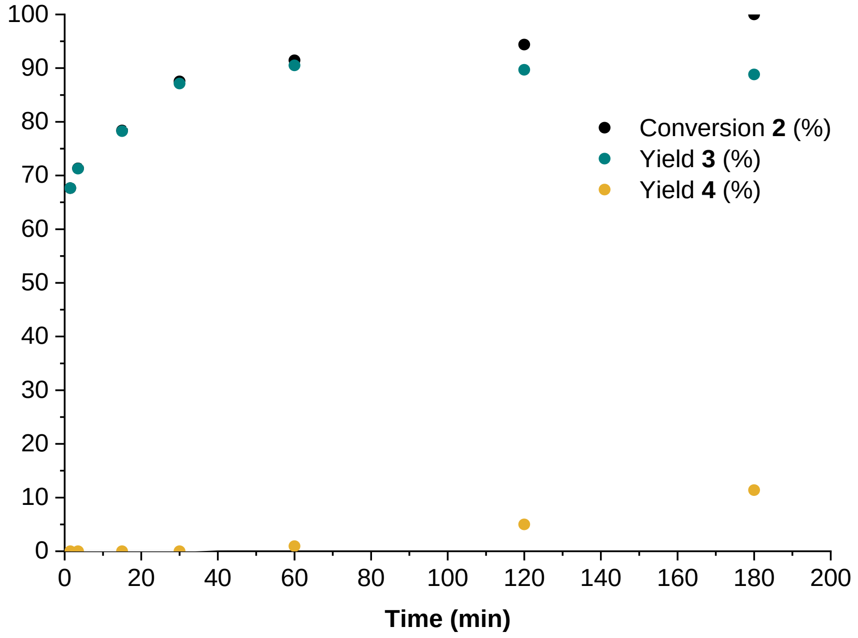

Figure S27: Conversion (**2**) and yields (**3, 4**) in hydroxymethylation using 11 eq. of HFIP.

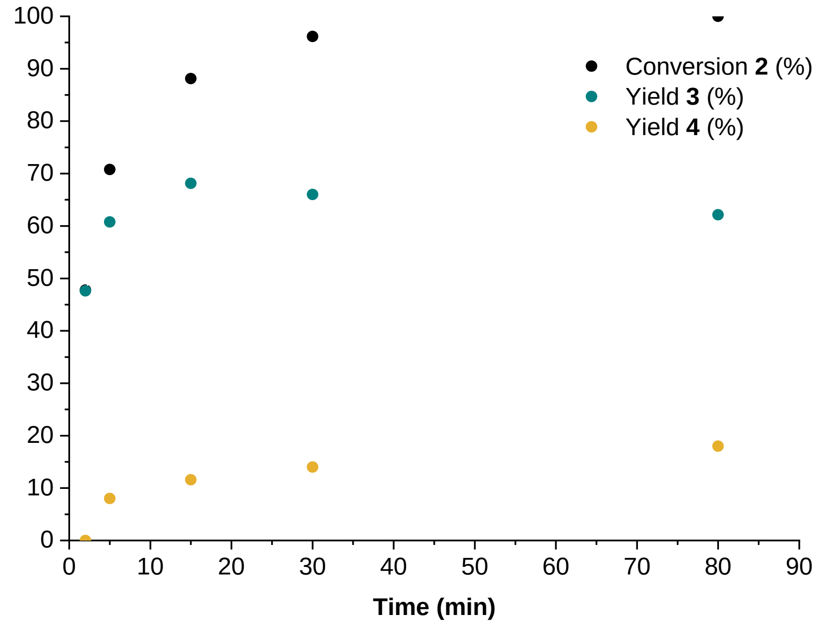

Figure S28: Conversion (**2**) and yields (**3, 4**) in hydroxymethylation using 6 eq. of HFIP.

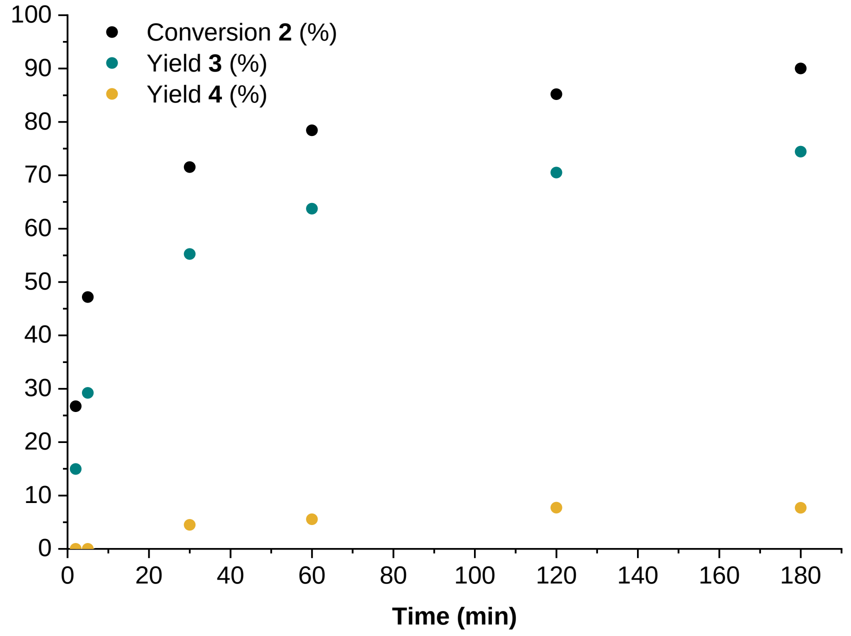

Figure S29: Conversion (**2**) and yields (**3, 4**) in hydroxymethylation using 3 eq. of HFIP.

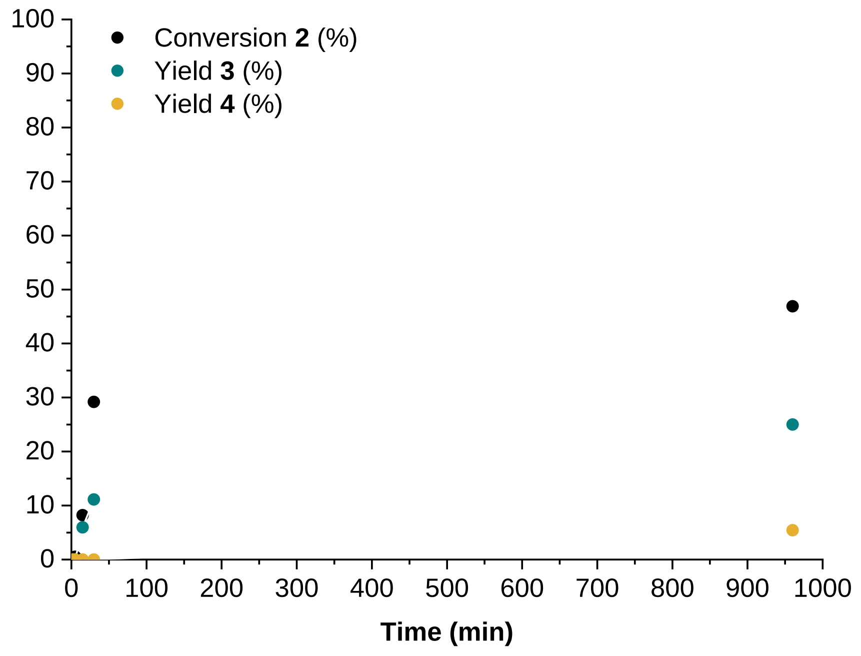

Figure S30: Conversion (**2**) and yields (**3, 4**) in hydroxymethylation using 0.5 eq. of HFIP.

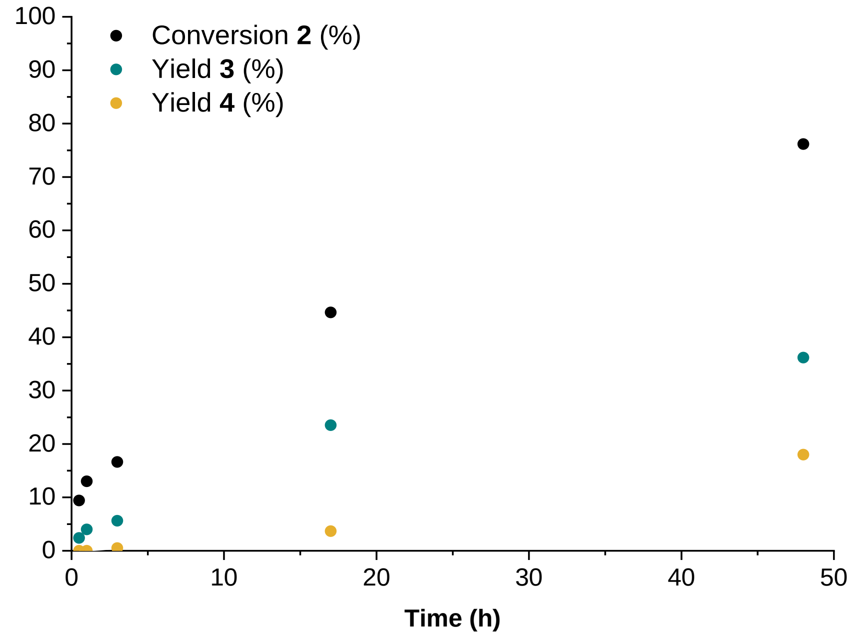

Figure S31: Conversion (**2**) and yields (**3, 4**) in hydroxymethylation using 0.05 eq. of HFIP.

10. Guaiacol-mediated hydroxymethylation

Reaction conditions: in a 2-mL round-bottom flask, furfural dimethylhydrazone **2** (1 eq.), formaldehyde (37% water, 1 eq.) and guaiacol were stirred at 50 °C.

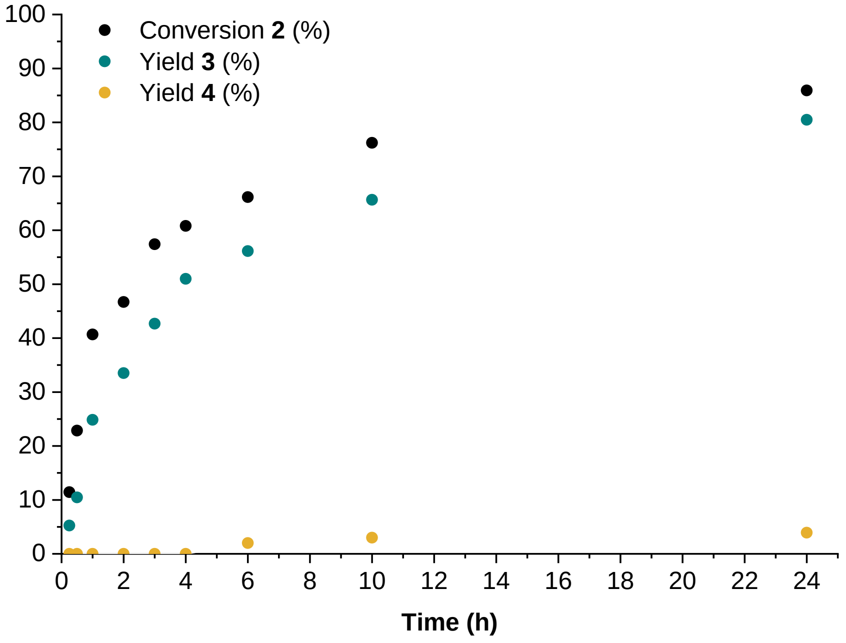

Figure S32: Conversion (**2**) and yields (**3, 4**) in hydroxymethylation using 12 eq. of guaiacol.

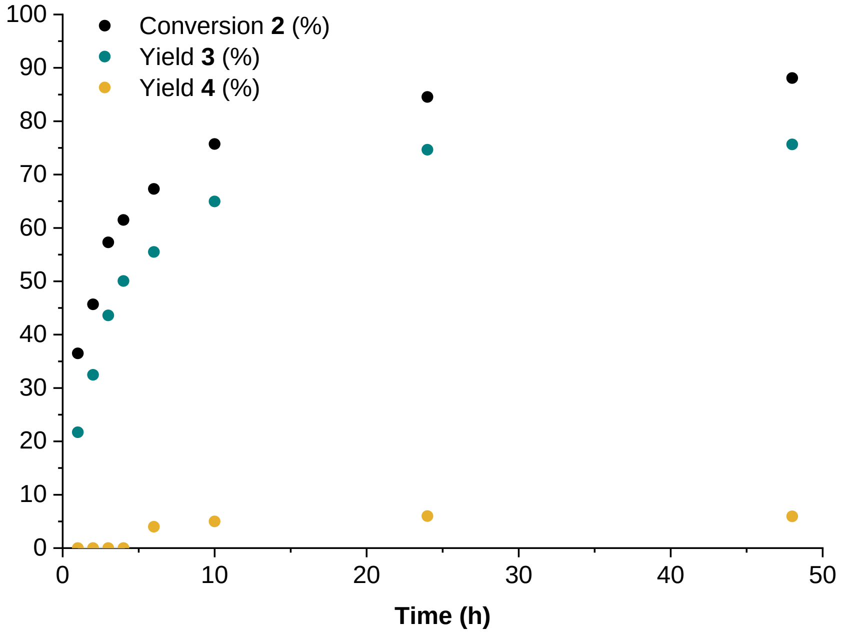

Figure S33: Conversion (**2**) and yields (**3, 4**) in hydroxymethylation using 6 eq. of guaiacol.

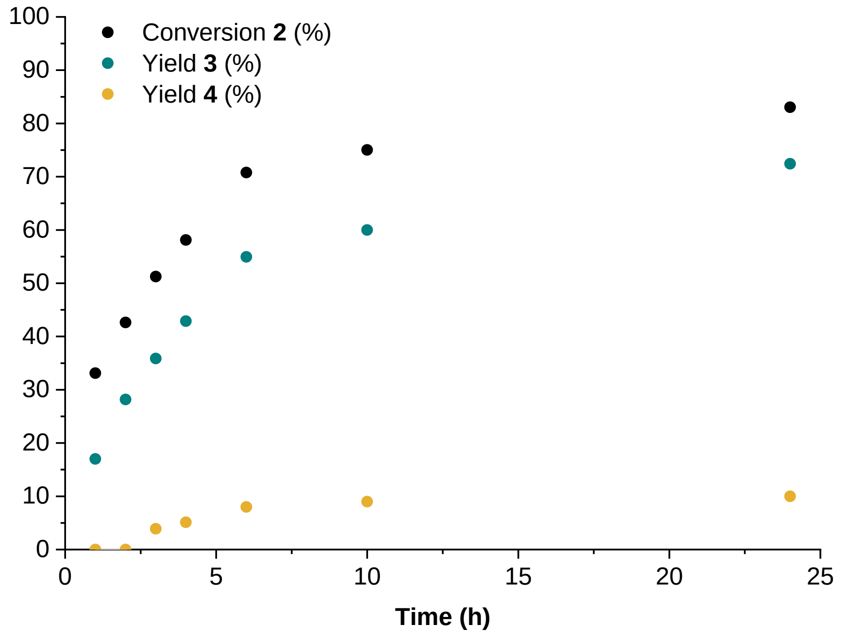

Figure S34: Conversion (**2**) and yields (**3, 4**) in hydroxymethylation using 3 eq. of guaiacol.

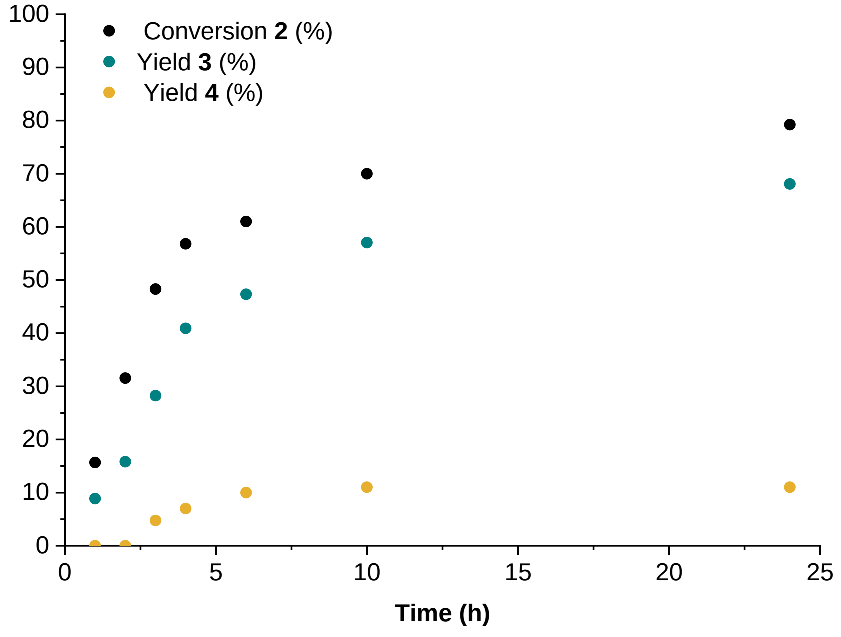

Figure S35: Conversion (**2**) and yields (**3, 4**) in hydroxymethylation using 1 eq. of guaiacol.

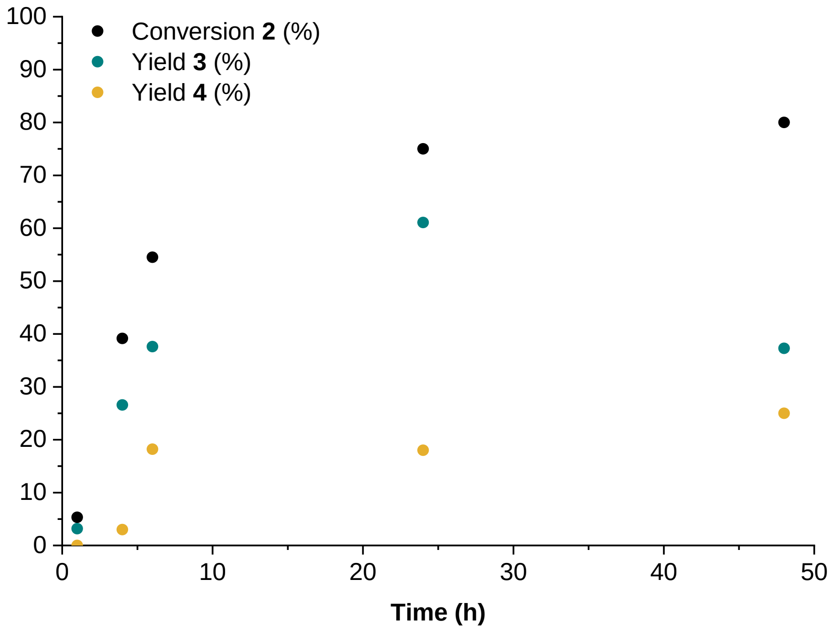

Figure S36: Conversion (**2**) and yields (**3, 4**) in hydroxymethylation using 0.5 eq. of guaiacol.

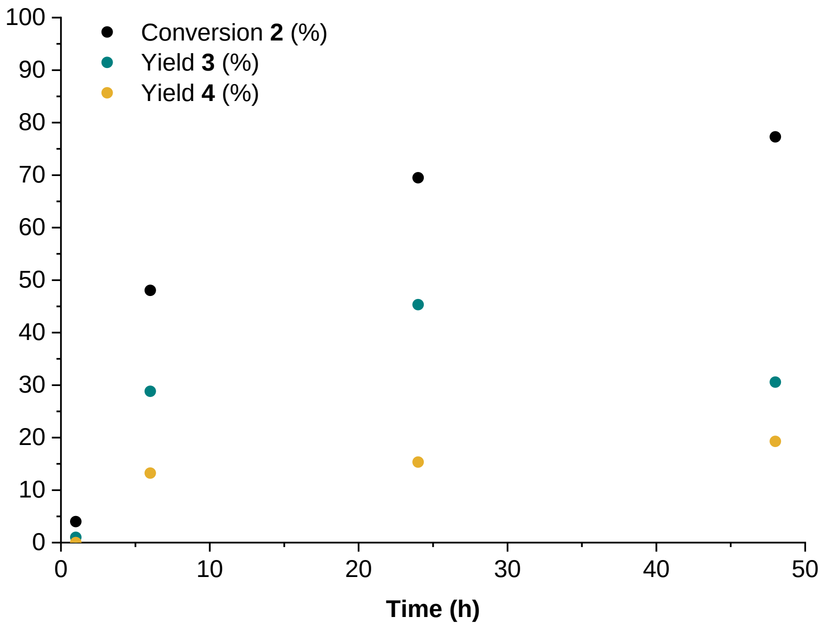

Figure S37: Conversion (**2**) and yields (**3, 4**) in hydroxymethylation using 0.3 eq. of guaiacol.

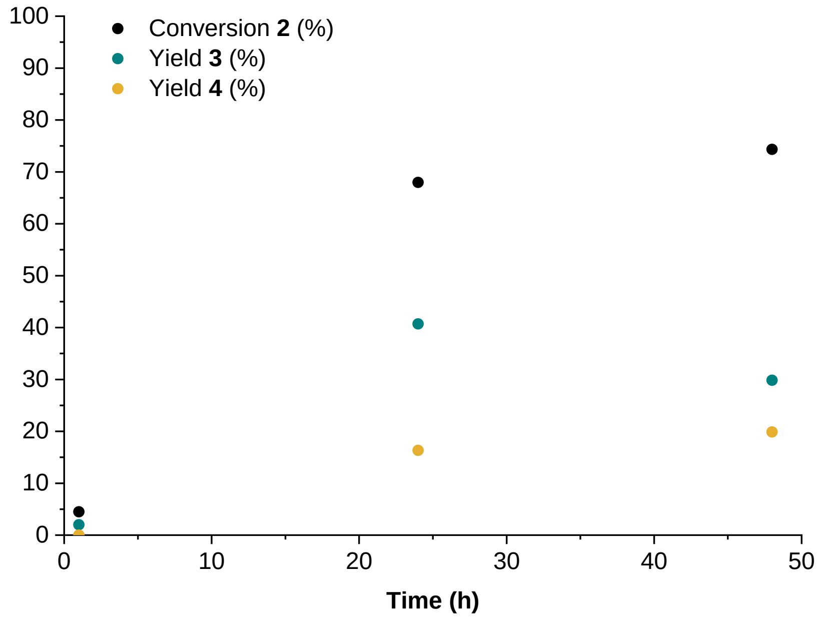

Figure S38: Conversion (**2**) and yields (**3, 4**) in hydroxymethylation using 0.2 eq. of guaiacol.

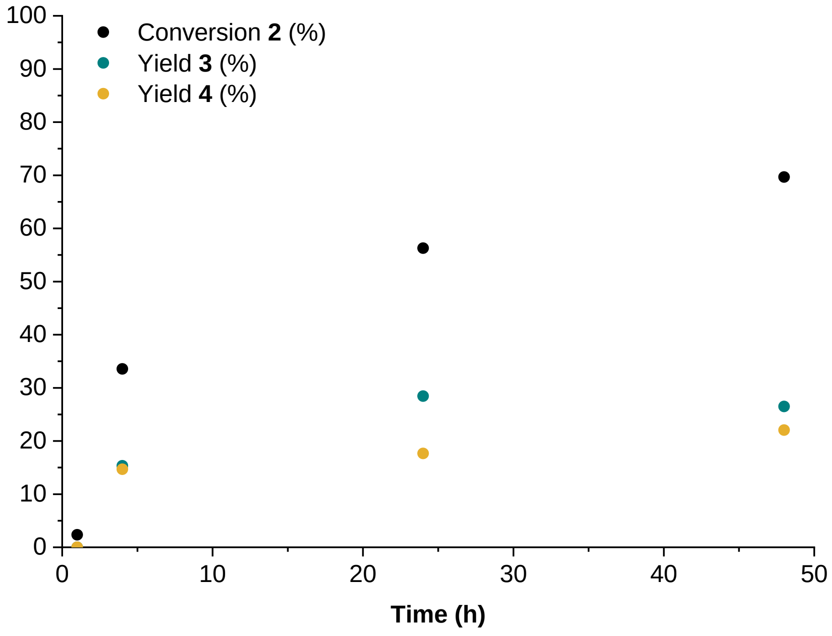

Figure S39: Conversion (**2**) and yields (**3, 4**) in hydroxymethylation using 0.1 eq. of guaiacol.

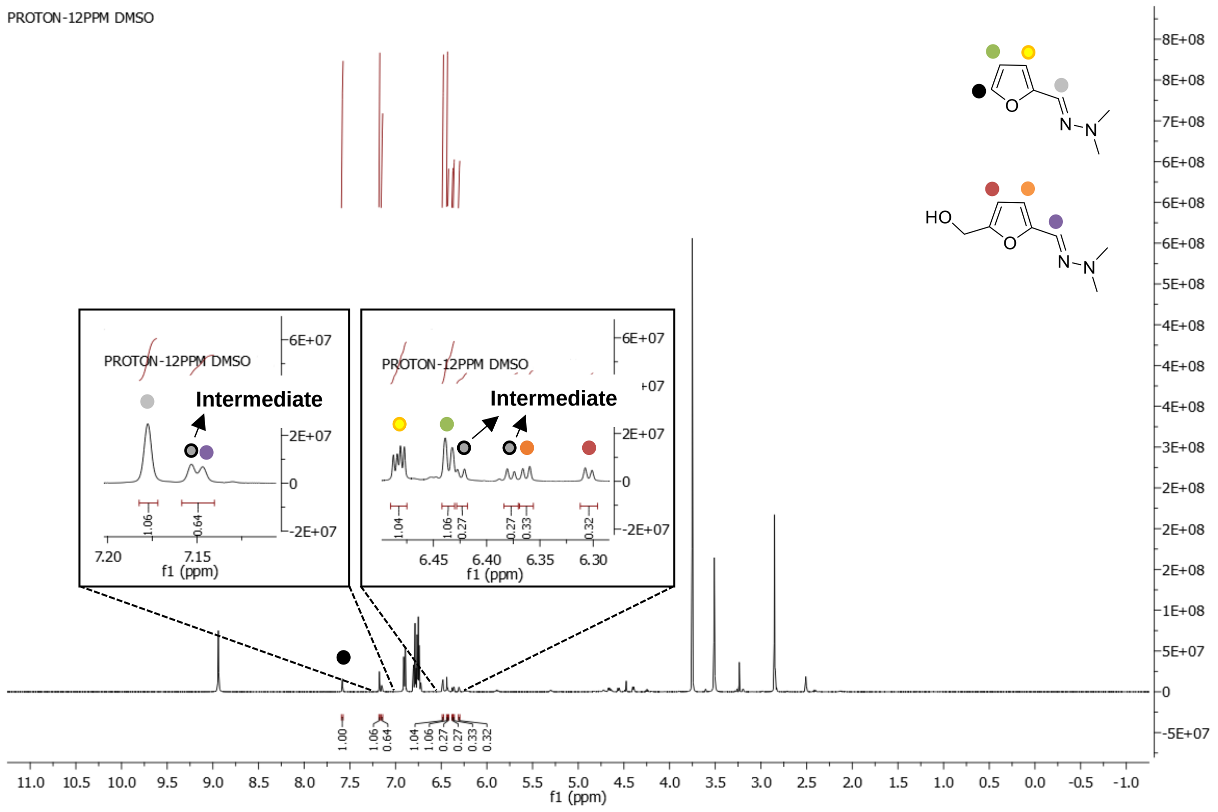

Figure S40: Resulting NMR spectra of the crude of (**2**) and formaldehyde in guaiacol (3 eq.) at 50 °C after 1 hour (grey and black circles: intermediate signals).

11. Computational details

All calculations were performed using Gaussian 16 rev A.01,[10] at the B3LYP/6-311++G(d,p) level of theory (in line with our previous work[11]). Solvation effects were included using the PCM formalism. We here used the solvent parameters for 2,2,2-trifluoroethanol. Optimisation were conducted without any constraint and systematically followed by frequencies calculations to confirm the nature of the stationary points.

In a first set of calculations, we optimised a pre-reacting complex consisting in a HCHO molecule, interacting with (**2**) and one molecule of HFIP (first structure provided hereafter). From this species, we then conducted a relaxed scan probing the formation of the C-N bond by addition of HCHO on hydrazone (24 steps of -0.05 Å, C-N distance ranging from 2.658 to 1.458 Å). This scan revealed a local maximum at 1.658 Å (relative energy of +6.0 kcal/mol) but more interestingly suggested the zwitterionic adduct could indeed be associated to a stable geometry. A full optimisation of the end point eventually confirmed this, additionnally revealing the full transfer of one proton from HFIP to the adduct (second geometry provided hereafter). From this geometry, two new starting geometries were produced replacing HFIP by methanol and water, and fully relaxed. In both cases, the adducts decomposed back into the separated reagents (HCHO, solvent, (**2**)), confirming the instability of the adducts in these conditions (third and fourth geometries).

In a second set of calculations, we then considered the transfer of the hydroxymethyl moiety from hydrazone group to the furanic ring of a second equivalent of (**2**). Starting from an optimised pre-reacting complex (seventh geometry), we then identified a transition state associated to the desired reaction process (eighth geometry). IRC calculation confirmed this transition state indeed connects the pre-reacting complex to the envisoned product (nineth geometry). Satisfactorily, the associated activation and reaction energies are rather comparable to those we previously observed for the hydroxymethylation of furanic ring via electrophilic aromatic substitution: +23.9 kcal and -5.9 kcal/mol, respectively (Gibbs' free energy at ambient temperature and pressure), ensuring the plausibility of the proposed mechanism.

Non covalent adduct HCHO/HFIP/(2) - E=-1362.16483380 a.u.

C	2.3795845372	2.5600537762	2.4940502915
C	2.4778985201	3.7059806074	3.2207863659
C	1.5252834905	4.6103381621	2.6602446941
C	0.9139395942	3.9445932745	1.6285764936
O	1.4352549133	2.6870027506	1.5231107404
H	2.8843017702	1.6096252495	2.535637645
H	3.1446462333	3.8841088666	4.0492288482
H	1.3176538896	5.6211942648	2.974409795
C	-0.1264317692	4.372026534	0.723520875
H	-0.4713686882	5.3935136578	0.8806886218
N	-0.5823961411	3.5953064208	-0.1949191424
N	-1.5195647527	4.0474372585	-1.0860087025
C	-2.2974507033	2.9357626768	-1.6340882817
H	-2.8127131989	3.2663480457	-2.5374552119
H	-1.6136071449	2.1239812983	-1.8783243896
H	-3.0402081962	2.5626329601	-0.9153958267
C	-2.2887894857	5.2481403018	-0.7716491964
H	-1.6419770388	6.1281117881	-0.7676715241
H	-3.0396371899	5.3907316668	-1.5473045339
H	-2.7906654294	5.164951282	0.2035631963
C	0.1291432379	4.9842434046	-2.9482778237
H	0.8398854116	4.356448193	-2.3922972824
H	-0.5147214716	4.4771947115	-3.6840597965
O	0.1103194985	6.1918343757	-2.8083743875
H	-0.9154497744	7.3975002681	-3.5996552294
O	-1.4490287272	8.176136018	-3.8795008789
C	-2.1429504349	7.9453001348	-5.0562779681
H	-2.6682230521	8.8672504733	-5.3222631638
C	-1.192907604	7.6450400769	-6.2371833821
C	-3.2421205924	6.8768970517	-4.8645084648
F	-1.853955581	7.5166324744	-7.4032877782
F	-0.3168949403	8.6526867879	-6.3767455196
F	-0.4825932259	6.5132067752	-6.0423484819
F	-3.988129791	6.6939180977	-5.9672226413
F	-4.0701859404	7.25449473	-3.8733401378
F	-2.7289660427	5.6730320648	-4.5141960115

Zwitterion-HFIP (6) - E=-1362.17008474

C	4.446653799	-2.8081770573	0.0356895293
C	3.32941885	-3.5422808921	-0.2357839835
C	2.2616989638	-2.6072700902	-0.3401905938
C	2.8066916075	-1.366013902	-0.1250771172
O	4.1518447433	-1.486645147	0.1071256262
H	5.4794244514	-3.0696873647	0.1970648623
H	3.2787758744	-4.6134803073	-0.3450997723
H	1.2220651943	-2.8109474399	-0.542000969
C	2.1776020066	-0.0750881977	-0.113684144
H	1.10615907	-0.1115685004	-0.3139610961
N	2.8672240897	0.9782174206	0.140986865
N	2.1767523748	2.2739977532	0.1116702509
C	2.5350062355	2.939035167	1.4093559255
H	2.1571604649	3.9598498978	1.3910766412
H	2.0699574415	2.3762612716	2.2131232592

H	3.6175270964	2.9281552665	1.5037238547
C	2.7796880183	3.0275337726	-1.0384273696
H	2.478719849	2.543499475	-1.9652725917
H	2.4245330302	4.055853841	-1.0101318107
H	3.860891878	2.9949501852	-0.9335305891
C	0.6106098622	2.3001168799	-0.0534241289
H	0.4132910359	3.368687746	-0.1729530381
H	0.4105578596	1.7832967705	-0.9958431155
O	-0.0262696172	1.7739801808	1.0186067764
H	-0.4630531722	0.857459904	0.7542744736
O	-0.8563087551	-0.4404733514	0.2784754398
C	-2.1007662487	-0.8091043352	-0.0975559855
H	-2.1666412133	-1.8661915812	-0.4176123837
C	-3.1081769452	-0.7104544995	1.0773672771
C	-2.5920919164	-0.0133625123	-1.3351957279
F	-4.3586449674	-1.1333406537	0.762868988
F	-2.6880422254	-1.4894512386	2.1002436111
F	-3.2312652829	0.543637304	1.5684546984
F	-3.8118667182	-0.3961279504	-1.7868987661
F	-1.728736309	-0.1989623751	-2.3614888326
F	-2.6553424252	1.3211215603	-1.1193350634

Adduct HCHO/MeOH/(2) - E=-687.881869059 a.u.

C	-3.4233093906	3.3650949801	-0.8354421122
C	-3.2422046115	3.6964607613	0.4712101549
C	-3.1946893859	2.462728861	1.1918740405
C	-3.351361252	1.4619500732	0.2679543344
O	-3.4923249052	2.0097201549	-0.9793352207
H	-3.5179170675	3.9394033667	-1.7417570315
H	-3.1522265402	4.6939339429	0.871251545
H	-3.0612630361	2.3284490152	2.2539175237
C	-3.3909119381	0.0281268635	0.4336851723
H	-3.263538045	-0.3127420214	1.4592629308
N	-3.5594119305	-0.7616181847	-0.5703463221
N	-3.5249549476	-2.1184607396	-0.3994315898
C	-3.6914875557	-2.6751178063	0.942013764
H	-3.7187622777	-3.7607937191	0.8616960777
H	-2.8464678279	-2.4020743214	1.5772350863
H	-4.6212277238	-2.3266671128	1.4129533907
C	-4.2684101899	-2.8062223033	-1.4568596551
H	-4.0062945361	-2.3593896498	-2.4150107322
H	-3.9917840443	-3.8611801941	-1.4626182883
H	-5.3535885646	-2.7240081769	-1.3111313445
C	-0.9808854631	-2.5802342212	-0.8325942588
H	-1.3202187485	-3.6225274234	-0.9282131618
H	-1.1454706871	-1.9257895352	-1.7009903757
O	-0.3753603513	-2.2003650968	0.1534650231
H	0.4418825429	-0.5177594601	0.3513574727
O	0.9234021048	0.3195201164	0.4706409673
C	2.3140572618	0.0649111653	0.2887815954
H	2.8418530216	1.0102704239	0.4245068971
H	2.6973282241	-0.653999511	1.0228096091
H	2.5325794552	-0.3116102771	-0.7176972725

Adduct HCHO/H2O/(2) - E=-648.578960302 a.u.

C	-2.6987141171	3.2220809122	0.8632393234
C	-2.883508338	3.7795870026	-0.3621260118
C	-3.1128866866	2.6952863277	-1.2671007906
C	-3.0546898884	1.5449220593	-0.5257373675
O	-2.7997667955	1.8600613749	0.7853501578
H	-2.4952750192	3.6228114551	1.8418851276
H	-2.8590637804	4.8324796359	-0.594215058
H	-3.299847675	2.755491946	-2.3278111534
C	-3.2140326992	0.1595871739	-0.8991366049
H	-3.3588311892	-0.0173603969	-1.9629061987
N	-3.1708244586	-0.7723970072	-0.0090713516
N	-3.2323001717	-2.0869069668	-0.3650948735
C	-3.7817476822	-2.9106216577	0.7122356903
H	-3.5581921052	-3.9591046007	0.5122178526
H	-3.3129362382	-2.6163370104	1.6502254564
H	-4.8693213409	-2.788568885	0.8036001448
C	-3.6577143981	-2.4506910418	-1.7140356248
H	-2.9516292667	-2.0696963651	-2.4548326472
H	-3.6738154217	-3.5369914665	-1.7864210159
H	-4.6599266378	-2.0624894706	-1.9471114776
C	-0.5291269359	-2.6167393172	0.0103286032
H	-0.6762093665	-3.5917042776	-0.4810079552
H	-0.5047148278	-1.7325276764	-0.6444147963
O	-0.3489348565	-2.5325019168	1.2084148944
H	-0.6273023164	-0.7977883553	2.0215360179
O	-0.8237125093	0.0790061954	2.3930734373
H	-1.573175188	0.4003840588	1.8736475111

Cation adduct HCHOH+/(2) (protonated zwitterion) - E=-572.477754818

C	-2.4325418151	3.6495070088	0.1766538974
C	-3.5922996145	3.9127165732	-0.4994747559
C	-4.18797377	2.653188888	-0.7515615982
C	-3.3448785152	1.7063836855	-0.2080349542
O	-2.2664561868	2.3231551522	0.362007354
H	-1.6551028104	4.2814147509	0.5745625874
H	-3.9653413746	4.8848503747	-0.7777932762
H	-5.1171862058	2.4610774114	-1.2661068308
C	-3.4553455229	0.2870361206	-0.1738846399
H	-4.3524032582	-0.1031013586	-0.6524111289
N	-2.5557871126	-0.4500425998	0.3837721022
N	-2.7685887641	-1.899380248	0.3796277716
C	-3.0456752244	-2.296768488	1.803860201
H	-3.0629073776	-3.3831127	1.8678408606
H	-2.2640755293	-1.8718668206	2.4300277988
H	-4.0108939474	-1.8844715125	2.0907088137
C	-3.819789789	-2.4650201977	-0.5300488464
H	-3.6500554747	-2.1029858452	-1.5428788063
H	-3.7166896822	-3.5479316628	-0.5072387247
H	-4.8082811384	-2.1814236158	-0.1746242352
C	-1.3781309484	-2.4374077944	-0.0641536489
H	-1.2656666784	-2.0872872954	-1.0896595345
H	-0.662179211	-1.9428992425	0.5903199898
O	-1.3538235023	-3.8147295213	-0.0240479041
H	-0.8338295466	-4.1381390627	0.7197165077

(2) - E=-457.555889827

C	2.5731321598	-2.6917806975	-1.1976356706
C	3.3883128997	-3.3206317337	-0.3099482319
C	4.0647456953	-2.2877406355	0.4132237906
C	3.6108338111	-1.0943596634	-0.088029378
O	2.694859018	-1.3354892892	-1.0775590302
H	1.8804306186	-3.0368542875	-1.9466942135
H	3.4957877964	-4.3866374927	-0.1854597436
H	4.7918799468	-2.4077398301	1.2010703774
C	3.9411752058	0.2653446213	0.2693735789
H	4.6826572236	0.3611286846	1.0601678766
N	3.3962177103	1.2710574198	-0.3309186174
N	3.7620157363	2.5395033685	-0.0392434044
C	2.7436383148	3.5208560141	-0.3995703967
H	3.2051848669	4.5052642136	-0.4895907963
H	2.3126490537	3.2394125296	-1.3594637506
H	1.9388587709	3.5745422577	0.3476800967
C	4.53952031	2.8226968215	1.1599081596
H	4.6971980036	3.8982642956	1.2227715246
H	4.0258337517	2.4834144289	2.0724234889
H	5.5172931068	2.3380909744	1.1096923397

Pre-reacting complex (2)/cation adduct HCHOH+&(2) - E=-1030.11096751

C	2.8885263608	-3.6353313986	0.0964342404
C	3.0459401299	-4.125142725	-1.1687405824
C	2.6792547944	-3.0699468017	-2.0453384081
C	2.323309294	-2.0072971258	-1.2485895956
O	2.4517433935	-2.3543945207	0.0699801503
H	3.0409872971	-4.0610252068	1.074542689
H	3.3812876162	-5.1143626625	-1.4344238753
H	2.6750607173	-3.0813892101	-3.1240927223
C	1.8734082775	-0.6993434171	-1.6037258221
H	1.7989339034	-0.5277485926	-2.6768593117
N	1.5849170623	0.1813053597	-0.7117707427
N	1.1129973527	1.4770933859	-1.2236452137
C	2.1253526809	2.1543631395	-2.1059354332
H	1.7444141108	3.1422532778	-2.3579096428
H	3.0578950163	2.2320471665	-1.5549253651
H	2.2582516119	1.5715167607	-3.0129840864
C	-0.2217119282	1.3479164641	-1.9071996525
H	-0.9003863921	0.8121895308	-1.2444211361
H	-0.5932008261	2.3502058878	-2.1144788327
H	-0.0897516613	0.8067634668	-2.8408303005
C	0.9139849099	2.3031220586	0.0680905081
H	0.5770443905	3.2881915236	-0.2466076299
H	0.1380299965	1.7734331744	0.6192451224
O	2.0974899356	2.4344580482	0.7570857566
H	2.3101474931	1.5894409441	1.1768068599
C	-2.9981195957	3.3311315844	1.0832226606
C	-3.2967966686	3.2527994728	2.4067777977
C	-3.2200502622	1.8669650347	2.7538024356
C	-2.8795319254	1.1925379403	1.6094150237
O	-2.7382821721	2.0863371763	0.5788627627
H	-2.9271836778	4.1463079399	0.3829352428
H	-3.5422959104	4.0784156348	3.0559290687

H	-3.3968069585	1.4230036871	3.7209654796
C	-2.6840678997	-0.2174889363	1.3684453035
H	-2.7982761038	-0.8485969446	2.247239171
N	-2.3887363441	-0.6682041825	0.1939398052
N	-2.143483049	-1.9850736071	-0.0049656395
C	-2.3791986047	-2.4006057087	-1.3845493697
H	-3.4481735148	-2.5519264877	-1.5921845454
H	-1.9995366228	-1.6293385421	-2.0529959142
H	-1.8485268355	-3.3340378296	-1.5768011877
C	-2.4883125199	-2.9571645441	1.0244105078
H	-2.2401423327	-3.9510660235	0.6553114581
H	-1.9101600475	-2.7774758748	1.9336258208
H	-3.5593074913	-2.9278463174	1.2765751449

Transition state - E=-1030.06926739

C	2.8215545607	4.3274374567	-2.8750743165
C	1.6499426708	5.0024096347	-2.7183816703
C	0.9887866383	4.3962826596	-1.6076526151
C	1.8092637169	3.3904609819	-1.1642763091
O	2.9367037103	3.3434176467	-1.9399160234
H	3.6419886971	4.4165831742	-3.5674050982
H	1.3020938785	5.8275863685	-3.3190006344
H	0.032719487	4.6654091326	-1.186492703
C	1.6472841486	2.4626529597	-0.0734192284
H	0.7154391815	2.5727074611	0.478005292
N	2.5476737886	1.5849313113	0.2059709532
N	2.3267079065	0.6580851804	1.2053122144
C	3.5998987867	0.2708624811	1.8261029649
H	3.4407205936	-0.6097538517	2.4486782724
H	4.3154334311	0.0368264109	1.0394188832
H	4.00097818	1.081858517	2.4452026813
C	1.2348953195	0.8784711232	2.1592698748
H	0.2684308002	0.8580528791	1.6533268705
H	1.2551246857	0.0714883778	2.8897587137
H	1.3455175312	1.8371985015	2.6822443732
C	1.7763934602	-1.2796258705	-0.2225893301
H	1.7008253899	-1.8388383052	0.6954288382
H	1.0226536769	-0.5743194143	-0.5347836337
O	2.9156444554	-1.3730802708	-0.8460993079
H	2.9622943178	-0.7777778294	-1.6109187947
C	0.354714144	-2.9710034619	-1.0515304056
C	-0.0326218182	-2.3796712619	-2.240716203
C	-1.2971706162	-1.804639171	-2.0237853881
C	-1.6533017901	-2.1080424869	-0.7157420114
O	-0.6672153751	-2.8397960833	-0.1335513059
H	1.0859072773	-3.7311226539	-0.8310357802
H	0.5531215624	-2.351755253	-3.146859978
H	-1.8867564508	-1.2301513543	-2.7201764559
C	-2.827470533	-1.7587108861	0.0253355849
H	-3.5687383323	-1.1740720493	-0.5115761022
N	-2.9571708925	-2.1400835239	1.2662658745
N	-4.0139070978	-1.8092225985	1.9741040619
C	-4.1423116842	-2.4296685772	3.2870645641
H	-4.8688005753	-3.2500670074	3.2664321913
H	-3.1693551218	-2.8202429338	3.5768648469

H	-4.4698914188	-1.6883284914	4.0188875465
C	-5.1515972799	-1.0988855633	1.4067646351
H	-5.8864992524	-0.9370888322	2.1921336874
H	-4.8413395464	-0.1275512302	1.010216524
H	-5.6153222119	-1.6771062969	0.5980838467

Product - E=-1030.11796410

C	0.9484124818	-3.4601656718	-1.0771965035
C	1.4597590274	-3.8675817099	0.1149390317
C	0.6287866821	-3.2883424074	1.1252917255
C	-0.3371693143	-2.5649687117	0.4734368975
O	-0.1455724562	-2.6651085632	-0.8795129389
H	1.2231996924	-3.6350280023	-2.1038520308
H	2.3187877799	-4.5040443846	0.2576584755
H	0.7267241006	-3.3941361043	2.1944184799
C	-1.4414426332	-1.7896761921	0.9891037788
H	-1.5039531995	-1.7549273379	2.0750559357
N	-2.2690388055	-1.1873992104	0.2019755968
N	-3.2589380107	-0.4055225038	0.6977583247
C	-4.3748776636	-0.2584120182	-0.2331550358
H	-4.964353129	0.6164371764	0.0450368981
H	-3.9760165189	-0.1180436649	-1.2369696
H	-5.0305572568	-1.140725934	-0.2333819189
C	-3.6100720568	-0.4801735951	2.1109804655
H	-2.7729527337	-0.1570571795	2.7339188942
H	-4.4476826013	0.1912562588	2.2930576301
H	-3.8982732331	-1.4993427985	2.4095561915
C	-1.6601601392	2.9400828228	0.5590010076
H	-1.2688793719	3.2145993177	1.5396746798
H	-1.9476172939	1.8853665388	0.5727858847
O	-2.7652266264	3.7868137617	0.2838804608
H	-3.3995613224	3.3183844365	-0.2689220367
C	-0.5346312372	3.1755723918	-0.4726528165
C	-0.752858242	2.6439475141	-1.844274713
C	0.1997188923	1.7384196128	-2.1299560624
C	1.0610837102	1.6431872301	-0.9768098676
O	0.6535463253	2.4621206805	-0.0190514463
H	-0.2837112754	4.241167586	-0.4831100353
H	-1.5592980347	2.9660467194	-2.4874453606
H	0.3270487405	1.1670799274	-3.0365356231
C	2.1797817978	0.8202248356	-0.8317154575
H	2.4451396475	0.1777539205	-1.6621049423
N	2.8532015546	0.8702789392	0.3173148939
N	3.8776719891	0.13527498	0.5138743127
C	4.5584673746	0.2590183874	1.8048953689
H	5.5940033186	0.563436417	1.6397790196
H	4.0350924576	1.0051140332	2.3958889914
H	4.5458514797	-0.706646624	2.3143715203
C	4.4045733684	-0.8185637074	-0.469794057
H	5.2671827346	-1.3158424879	-0.0358763903
H	3.6397510761	-1.560120869	-0.7138476383
H	4.7071309244	-0.2894468099	-1.3764289906

12. References

[1] F., A.W.L., and Lin, C.C.L. (2009) *Purification of laboratory chemicals (6th ed.)*, Butterworth-Heinemann.

[2] (a) Vold, R.L., Waugh, J.S., Klein, M.P., and Phelps, D.E. (1968) Measurement of spin relaxation in complex systems. *The Journal of Chemical Physics*, **48** (8), 3831–3832. (b) Freeman, R., and Hill, H.D. (1969) High-resolution studies of nuclear spin–lattice relaxation. *The Journal of Chemical Physics*, **51** (7), 3140–3141.

[3] Trindade, I.B., and Louro, R.O. (2020) Introduction to biomolecular nuclear magnetic resonance and metals. *Practical Approaches to Biological Inorganic Chemistry*, 155–199.

[4] Quantitative NMR - Technical Details and TraceCERT® Certified Reference Materials- milliporesigma.

[5] Time of Flight the Power of Accurate Mass. https://www.agilent.com/Library/slidepresentation/Public/ASTS-2014_TOF_Power_of_Accurate_Mass.pdf (accessed 2023-07-18).

[6] V. Karaluka, K. Murata, S. Masuda, Y. Shiramatsu, T. Kawamoto, H. C. Hailes, T. D. Sheppard, A. Kamimura, "Development of a microwave-assisted sustainable conversion of furfural hydrazones to functionalised phthalimides in ionic liquids", *RSC Adv.* **2018**, *8*, 22617–22624.

[7] Common_Solvents_for_Organic_Reactions, assessed on December, 20th 2024.

[8] A. Charkhesht, D. Lou, B. Sindle, C. Wen, S. Cheng, N. Q. Vinh, *J. Phys. Chem. B* **2019**, *123*, 8791–8799.

[9] G. Fan, C. Liao, T. Fang, S. Luo, G. Song, *Carbohydrate Polymers* **2014**, *112*, 203–209.

[10] Gaussian 16, Revision C.01, M. J. Frisch, G. W. Trucks, H. B. Schlegel, G. E. Scuseria, M. A. Robb, J. R. Cheeseman, G. Scalmani, V. Barone, G. A. Petersson, H. Nakatsuji, X. Li, M. Caricato, A. V. Marenich, J. Bloino, B. G. Janesko, R. Gomperts, B. Mennucci, H. P. Hratchian, J. V. Ortiz, A. F. Izmaylov, J. L. Sonnenberg, D. Williams-Young, F. Ding, F. Lipparini, F. Egidi, J. Goings, B. Peng, A. Petrone, T. Henderson, D. Ranasinghe, V. G. Zakrzewski, J. Gao, N. Rega, G. Zheng, W. Liang, M. Hada, M. Ehara, K. Toyota, R. Fukuda, J. Hasegawa, M. Ishida, T. Nakajima, Y. Honda, O. Kitao, H. Nakai, T. Vreven, K. Throssell, J. A. Montgomery, Jr., J. E. Peralta, F. Ogliaro, M. J. Bearpark, J. J. Heyd, E. N. Brothers, K. N. Kudin, V. N. Staroverov, T. A. Keith, R. Kobayashi, J. Normand, K. Raghavachari, A. P. Rendell, J. C. Burant, S. S. Iyengar, J. Tomasi, M. Cossi, J. M. Millam, M. Klene, C. Adamo, R. Cammi, J. W. Ochterski, R. L. Martin, K. Morokuma, O. Farkas, J. B. Foresman, and D. J. Fox, Gaussian, Inc., Wallingford CT, 2016.

[11] S. Behloul, O. Gayraud, G. Frapper, F. Guégan, K. Upitak, C. Thomas, Z. Yan, K. de Oliveira Vigier, F. Jérôme, “Acid-Catalyzed Activation and Condensation of the =C5H Bond of Furfural on Aldehydes, an Entry Point to Biobased Monomers”, *ChemSusChem* **2024**, *17*, e202400289.
